# 2,4-D attenuates salinity-induced toxicity by mediating anatomical changes, antioxidant capacity and cation transporters in the roots of rice cultivars

**DOI:** 10.1038/s41598-017-09708-x

**Published:** 2017-09-05

**Authors:** Faisal Islam, Muhammad A. Farooq, Rafaqat A. Gill, Jian Wang, Chong Yang, Basharat Ali, Guang-Xi Wang, Weijun Zhou

**Affiliations:** 1 Institute of Crop Science and Zhejiang Key Laboratory of Crop Germplasm, Zhejiang University, Hangzhou, 310058 China; 2 Institute of Pure and Applied Biology, Bahauddin Zakariya University, Multan, Pakistan; 3 Institute of Crop Science and Resource Conservation, University of Bonn, 53115 Bonn, Germany; 4 Department of Environmental Bioscience, Meijo University, Nagoya City, Aichi 468-8502 Japan

## Abstract

Growth regulator herbicides are widely used in paddy fields to control weeds, however their role in conferring environmental stress tolerance in the crop plants are still elusive. In this study, the effects of recommended dose of 2,4-dichlorophenoxyacetic acid (2,4-D)  on growth, oxidative damage, antioxidant defense, regulation of cation transporter genes and anatomical changes in the roots of rice cultivars XS 134 (salt resistant) and ZJ 88 (salt sensitive) were investigated under different levels of saline stress. Individual treatments of saline stress and 2,4-D application induced oxidative damage as evidenced by decreased root growth, enhanced ROS production, more membrane damage and Na^+^ accumulation in sensitive cultivar compared to the tolerant cultivar. Conversely, combined treatments of 2,4-D and saline stress significantly alleviated the growth inhibition and oxidative stress in roots of rice cultivars by modulating lignin and callose deposition, redox states of AsA, GSH, and related enzyme activities involved in the antioxidant defense system. The expression analysis of nine cation transporter genes showed altered and differential gene expression in salt-stressed roots of sensitive and resistant cultivars. Together, these results suggest that 2,4-D differentially regulates the Na^+^ and K^+^ levels, ROS production, antioxidant defense, anatomical changes and cation transporters/genes in roots of rice cultivars.

## Introduction

Crop plants growing under field conditions frequently face a variety of stresses that can cause severe yield losses. Among the environmental stressors, salinity is one of the most devastating abiotic stresses in rice, and salt-affected soils currently account for approximately 20% of the total paddy planting area^1, 2^. More seriously, the area of salt-affected land is expanding and spreading not only in China but also in different regions of the world^1–5^. High saline stress affects plant growth by modifying the morpho-physiological and biochemical traits of the exposed plants. Consequently, plants must activate different adaptive features at both the physiological and molecular levels because they show altered anatomical and physiological mechanisms, thus enabling the stressed plants to grow well and maintain their reproductive capacity^6–10^. These mechanisms include the regulation of hormones, the adjustment of photosynthesis and respiratory metabolism, the modulation of antioxidant defenses and the efflux of toxic ions. Furthermore, salinity imposes osmotic stress and ion toxicity leading to nutrient imbalances and the production of reactive oxygen species (ROS)^7–9^.

Herbicides are extensively used on agricultural fields to control weed growth around the world. The application of pre/post emergence herbicides often affects crop physiology by hindering photosynthesis, altering stomatal closure and changing metabolic or physiological functions^11–15^. The herbicide-induced stress may be short-term or long-lasting. Among herbicides, growth regulator herbicides, such as 2,4-dichlorophenoxyacetic acid (2,4-D) are widely used as pre/post emergence systemic herbicides belonging to the class of phenoxy herbicides^16, 17^. The 2,4-D was the first commercial herbicide to be introduced -on the market for the control of broadleaf weeds and plants^12, 16, 17^. The 2,4-D herbicide remains one of the most commonly used herbicides in the world because of its low cost, selectivity, efficacy and broad spectrum of weed control^16, 17^. The physiological responses of sensitive plants to auxinic herbicides include abnormal growth, senescence, and plant death^17–20^. The rice plants are also susceptible to the 2,4-D injury like wheat, barley, soybean, peanut and cotton^12, 13, 15, 16^. These symptoms may include stem and leaf twisting, head malformations, floret sterility, root malformation and inhibition^16, 20–23^. Additionally, 2,4-D induces the overproduction of reactive oxygen species (ROS) and lipid peroxidation^19, 24, 25^. Because of its widespread use, 2,4-D is a frequently detected herbicide in the atmosphere, surface and ground waters via percolation from agricultural lands^26–30^. However, there is little information about how this growth regulator herbicide affects crop growth and physiology under biotic/abiotic stress conditions. At low concentrations, 2,4-D acts like a mimicker of natural auxin, promoting cell division and elongation.

Rice (*Oryza sativa* L.) is a staple food for a large global population and is grown in both tropical and temperate regions of the world. Among cereals, rice (*O. sativa*) is also considered to be the most salt-sensitive crop. Therefore investigating different strategies to make rice plants more tolerant and to improve its productivity under salinity is an important challenge for researchers to cope with reduced food production due to soil salinization^3, 4, 31^. However, the question about a how recommended dose of 2,4-D regulates salt tolerance in the rice plants is still left to be resolved. Because roots are the first organ of a plant to have contact with salinity/herbicide (often 2,4-D is directly applied onto the soil by mixing with sand or in the form of granules), the hypothesis that 2,4-D application can influence plant root growth and morphology, thereby affecting the uptake of cations (Na^+^ and K^+^) and modulating enzymatic and non-enzymatic antioxidants, seems reasonable. Furthermore, it would be interesting to determine how 2,4-D regulates root growth in contrasting rice cultivars at different levels of salinity. The resolution of these questions will provide information about whether and how the application of growth regulator herbicide (2,4-D) significantly confers salt tolerance to rice plants. The results were quantified by measuring several morph-physiological parameters. To investigate the changes at the molecular level, we  have also evaluated the gene expression profiling of antioxidant enzymes and cation transporters in the roots of rice cultivars under controlled and stress conditions.

## Results

### Effects of 2,4-D/salt on biomass production, K^+^ and Na^+^ accumulation

Effects of individual and combined stresses of herbicide and salt on root biomass (fresh and dry weight), Na^+^ and K^+^ concentration are shown in Table 1. Exposure of rice cultivars to saline stress treatments effects plant growth, which causes a significant decrease in dry (DW) and fresh weights (FW) in a dose dependent manner. Under severe salt stress treatment (T4), the decrease in FW and DW was 39% and 48﻿% respectively in the ZJ 88 cultivar, while 31% and 38% decrease respectively was in the XS 134 cultivar compared to the control plants (Table 1). The herbicide (2,4-D) application alone (T2) also reduced FW and DW more in the ZJ 88 cultivar than in XS 134. The results of plant biomass indicated that salt stress effects more severely than herbicide treatment (T2). However, when plants were exposed to a combined stress of herbicide + mild salt treatment (T5), considerable increase in root biomass was noted compared to mild salt treatment (T3) in both rice cultivars. Conversely, under herbicide + severe salt treatment (T6), the ZJ 88 cultivar displayed a significant reduction in DW (50%) and FW (41%) compared with the control, while slight improvement in FW and DW of the XS 134 cultivar roots was observed when compared to its respective salt stress treatment (T4) (Table 1).

**Table 1:** Effects of individual and combined stresses of 2,4-D and salinity on plant biomass, Na^+^ and K^+^ accumulation in the roots of two rice cultivars.

Cultivar	Treatment	Fresh weight of plant (g)	Dry weight of plant (g)	Na^+^ (mmol g^−1^ DW)	K^+^ (mmol g^−1^ DW)	K^+^/Na^+^ ratio
ZJ 88	Control (CK)	5.45 ± 0.39ab (100%)	0.80 ± 0.05abcd (100%)	3.60 ± 0.20fg (100%)	4.16 ± 0.60bc (100%)	1.18 ± 0.22bc (100%)
	T2	5.02 ± 0.39abcd (−8%)	0.65 ± 0.03cde (−19%)	3.50 ± 0.06fg (−3%)	4.00 ± 0.29bc (−4%)	1.14 ± 0.07bc (−3%)
	T3	4.30 ± 0.36cde (−21%)	0.63 ± 0.07cde (−21%)	5.90 ± 0.10bc (64%)	2.10 ± 0.18fg (−50%)	0.36 ± 0.04ghi (−69%)
	T4	3.30 ± 0.32fg (−39%)	0.42 ± 0.06ef (−48%)	8.85 ± 0.29a (146%)	1.07 ± 0.23 h (−75%)	0.13 ± 0.01i (−89%)
	T5	4.80 ± 0.17abcd (−12%)	0.72 ± 0.04cde (−10%)	4.60 ± 0.25de (28%)	3.10 ± 0.22de (−25%)	0.67 ± 0.03de (−43%)
	T6	3.20 ± 0.23 g (−41%)	0.40 ± 0.05 f (−50%)	8.03 ± 0.58a (123%)	1.51 ± 0.38gh (−64%)	0.19 ± 0.02hi (−84%)
XS 134	Control (CK)	5.76 ± 0.34a (100%)	0.95 ± 0.10a (100%)	3.30 ± 0.32 h (100%)	5.11 ± 0.75a (100%)	1.55 ± 0.17a (100%)
	T2	5.33 ± 0.40ab (−7%)	0.86 ± 0.07ab (−9%)	3.42 ± 0.31fg (4%)	4.70 ± 0.30ab (−8%)	1.37 ± 0.16ab (−11%)
	T3	4.80 ± 0.25bcd (−17%)	0.74 ± 0.05abcd (−22%)	5.05 ± 0.29 cd (53%)	2.81 ± 0.33ef (−44%)	0.56 ± 0.04fg (−64%)
	T4	4.00 ± 0.32efg (−31%)	0.59 ± 0.08def (−38%)	7.20 ± 0.47ab (118%)	2.00 ± 0.23fg (−60%)	0.28 ± 0.02ghi (−82%)
	T5	5.00 ± 0.20abcd (−13%)	0.84 ± 0.08abc (−12%)	4.13 ± 0.30ef (25%)	3.80 ± 0.25 cd (−26%)	0.92 ± 0.09 cd (−41%)
	T6	4.50 ± 0.53bcd (−22%)	0.67 ± 0.09cde (−29%)	6.50 ± 0.32bc (97%)	3.00 ± 0.29de (−40%)	0.46 ± 0.02fgh (−70%)

Average ± standard error from four separate replicates. Values with different letters are significantly different at *P* ≤ *0.05* as determined by Duncan’s test. CK = control, T2 = Recommended dose of herbicide (2,4-D), T3 = 4 dS m^−1^, T4 = 8 dSm^−1^, T5 = 4 ds m^−1^ + Recommended dose of herbicide, and T6 = 8 dS m^−1^  + Recommended dose of herbicide. FW = Fresh weight, DW = Dry weight, g = grams, dS m^−1^ = deciSiemens per meter.

Generally, herbicide alone treatment (T2) did not affect the uptake of Na^+^ and K^+^ in rice cultivars under control conditions. Whereas, with the increase in external NaCl concentration, Na^+^ concentration in roots was significantly increased in both cultivars, and the sensitive cultivar (ZJ 88) possessed a relatively higher Na^+^ concentration under mild (T3) and severe (T4) saline stress treatments than the resistant cultivar (XS 134). On the other hand, herbicide + mild salt treatment (T5) decreased Na^+^ accumulation (22% = ZJ 88, 18% = XS 134) and enhanced K^+^ content in both rice cultivars compared with their respective salt stress treatment (T3) (Table 1). Under herbicide + severe salt treatment (T6), the XS 134 cultivar maintained a significantly lower level of Na^+^ accumulation as compared to the ZJ 88 cultivar, which maintained a similar level of Na^+^ uptake compared to its respective salt stress treatment (T4). Regarding K^+^, there was a significant cultivar difference: cultivar ZJ 88 had a lower K^+^ content under all stress treatments than cultivar XS 134 (Table 1). In rice cultivars, K^+^/Na^+^ ratio decreased with increase of NaCl levels, but the XS 134 cultivar maintained higher K^+^/Na^+^ ratios under various stress treatments. Likewise, addition of a herbicide considerably improved the K^+^/Na^+^ ratio under mild stress conditions (T5), but under herbicide + severe salt treatment (T6), the K^+^/Na^+^ ratio was only improved in the XS 134 cultivar compared to its respective salt stress treatment (T4). In addition to this, the herbicide (2,4-D) treatment alone did not show any obvious change in Na^+^, K^+^ contents and K^+^/Na^+^ ratio compared to control (Table 1).

### Effects of 2,4-D/salt on oxidative stress

**Figure 1:**
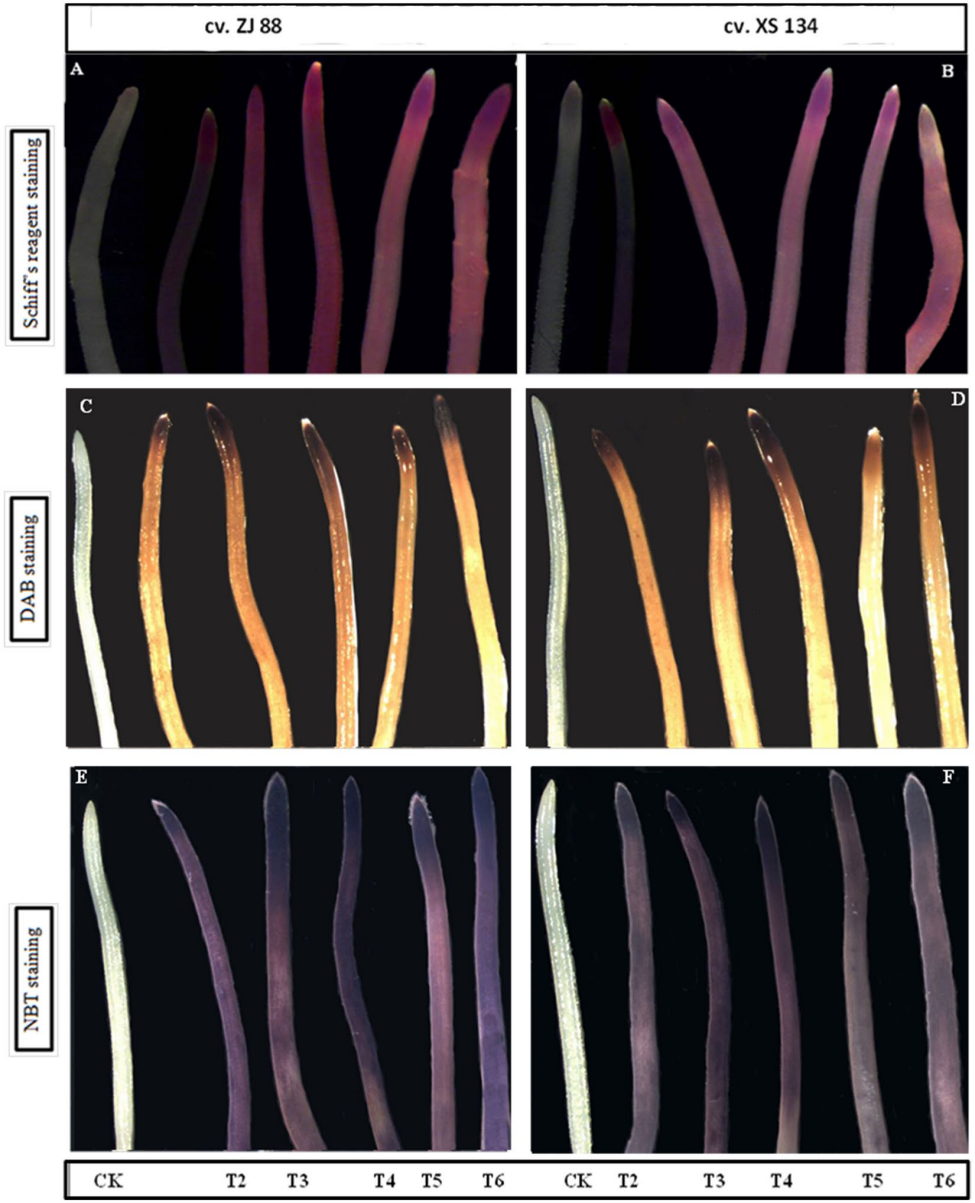
Histochemical detection of lipid peroxidation by Schiff’s reagent in roots of rice cultivars under light microscope (**A**) ZJ 88 and (**B**) XS 134. Bright fluorescence at root tips shows accumulation of aldehydes. Histochemical detection of (**C**,**D**) H_2_O_2_ and (**E** and **F**) O_2_^−^ by DAB and NBT in rice cultivars ZJ 88 (**C**,**E**) and XS 134 (**D,F**) roots, respectively. Brown color shows H_2_O_2_ accumulation, while blue coloration represents O_2_^−^ production. 20-day-old rice seedlings were treated as control (CK) with EC 1.2 dS m^−1^), T2 (recommended dose of 2,4-D), T3 (EC 4 dS m^−1^), T4 (EC 8 dS m^−1^), T5 (EC 4 dS m^−1^ + recommended dose of 2,4-D), T6 (EC 8 dS m^−1^ + recommended dose of 2,4-D) for 15 days Afterwards, the roots were stained with Schiff’s reagent, 3,3′-diaminobenzidine (DAB) or nitroblue tetrazolium (NBT), respectively, and immediately photographed under a light microscope (LEICA MZ 95 microscope equipped with LECIA DFC 300 FX camera). The experiment was repeated four times with similar results. Scale bar = 2 mm.

The malondialdehyde (MDA) content is widely used as an indicator of lipid peroxidation and electrolyte leakage (EL) is used to assess membrane integrity under stress conditions. Significant increases in MDA and EL contents were observed in both rice cultivars under mild (T3) and severe (T4) saline stress conditions as compared to their respective controls. Under combined stress conditions (T5 and T6), MDA content was decreased up to 12% and 18%, while EL was reduced up to 38% and 32% in resistant cultivar compared with its respective saline stress treatment T3 and T4, respectively. Whereas, in the cultivar ZJ 88, application of 2,4-D reduced MDA content up to 15% under mild saline + 2,4-D treatment (T_5_), and no significant change was found under severe saline + 2,4-D treatment (T_6_) as compared to their respective salt stress treatment (T4) (Table 2). Schiff’s reagent staining was used for *in situ* detection of salt/2,4-D induced lipid peroxidation in both rice cultivars roots. Under control conditions, negligible Schiff’s reagent staining was observed on the roots. However, under herbicide (T2) and salt stress treatments (T3 and T4), clear differences in the accumulation of aldehydes were detected in the roots of the ZJ 88 cultivar followed by the XS 134 cultivar. In combined stress treatments (T5 and T6), Schiff’s reagent staining was more pronounced in the ZJ 88 cultivar roots as compared to the XS 134 cultivar (Fig. 1A and B). But, the intensity of staining in combined treatments was relatively less than individual stress treatments (Fig. 1).

**Table 2:** Effects of individual and combined stresses of 2,4-D and salinity on malonaldehyde (MDA), hydrogen peroxide (H_2_O_2_), superoxide anion (O_2_^−·^) and electrolyte leakage (EL), superoxide dismutase (SOD), catalase (CAT), peroxidase (POD), ascorbate peroxidase (APX) in the roots of two rice cultivars.

Cultivar	Treatment	MDA (nmol g^−1^FW)	H_2_O_2_ (mmol g^−1^ FW)	O^.−^ (nmol min^−1^ g^−1^ FW)	EL (%)	SOD (U mg^−1^ protein)	POD (U mg^−1^ protein)	CAT (U mg^−1^ protein)	APX (U mg^−1^ protein)
ZJ 88	Control (CK)	1.53 ± 0.19e (100%)	22.34 ± 2.65hi (100%)	2.30 ± 1.40 f (100%)	14.20 ± 2.84 f (100%)	50.54 ± 2.90gh (100%)	54.19 ± 1.43 cd (100%)	98.73 ± 4.06ef (100%)	25.15 ± 1.15e (100%)
	T2	4.80 ± 0.29 cd (214%)	35.45 ± 3.53cde (59%)	3.20 ± 2.18de (29%)	25.31 ± 2.57def (78%)	90.49 ± 4.69bc (79%)	50.23 ± 2.89d (7%)	113.30 ± 7.02cde (15%)	28.82 ± 2.00dce (15%)
	T3	6.68 ± 0.68b (337%)	39.78 ± 3.51bc (78%)	4.08 ± 3.16bc (77%)	43.57 ± 4.91c (207%)	86.60 ± 4.36 cd (71%)	65.75 ± 2.22ab (21%)	109.60 ± 6.06def (11%)	29.82 ± 1.64de (19%)
	T4	9.73 ± 0.83a (536%)	45.63 ± 1.81a (104%)	4.90 ± 3.82a (113%)	70.39 ± 5.73ab (396%)	110.48 ± 4.81a (119%)	71.60 ± 3.86ab (32%)	100.40 ± 10.83ef (2%)	33.82 ± 2.34cde (34%)
	T5	5.67 ± 0.45bc (271%)	34.30 ± 2.70efg (54%)	3.60 ± 2.85 cd (57%)	34.41 ± 3.58cde (142%)	84.89 ± 4.84 cd (68%)	69.52 ± 2.35ab (28%)	130.70 ± 6.00bc (32%)	31.49 ± 1.47bcd (25%)
	T6	8.53 ± 0.90a (458%)	41.59 ± 3.20ab (52%)	4.43 ± 3.92ab (93%)	75.56 ± 5.42a (432%)	99.37 ± 6.27ab (97%)	80.63 ± 5.88a (49%)	90.33 ± 5.83 f (−9%	32.82 ± 1.25bc (30%)
XS 134	Control (CK)	1.57 ± 0.18e (100%)	20.41 ± 2.58i (100%)	2.37 ± 0.95 f (100%)	12.78 ± 1.59 f (100%)	45.87 ± 2.32 h (100%)	63.77 ± 2.43ab (100%)	101.67 ± 4.48def (100%)	24.78 ± 0.72e (100%)
	T2	3.47 ± 0.20d (121%)	24.82 ± 1.48hi (22%)	2.83 ± 2.45ef (19%)	19.97 ± 1.80 f (56%)	60.52 ± 2.95fg (32%)	64.89 ± 5.34ab (2%)	121.48 ± 3.38 cd (19%)	38.23 ± 2.60a (54%)
	T3	4.07 ± 0.49 cd (159%)	29.78 ± 3.06gh (46%)	3.43 ± 2.56 cd (45%)	35.41 ± 4.88 cd (177%)	63.82 ± 3.15ef (39%)	65.19 ± 2.22ab (2%)	132.13 ± 4.33bc (30%)	27.45 ± 1.15de (11%)
	T4	6.58 ± 0.64b (319%)	36.30 ± 1.98 cd (78%)	4.40 ± 3.26ab (86%)	60.27 ± 6.03b (372%)	89.86 ± 4.34bc (96%)	66.60 ± 4.86b (4%)	162.20 ± 5.13b (60%)	30.49 ± 1.56bc (23%)
	T5	3.57 ± 0.18d (127%)	25.38 ± 2.00gh (24%)	3.03 ± 2.64de (28%)	22.04 ± 2.51ef (72%)	52.34 ± 2.45fgh (14%)	64.82 ± 2.93ab (2%)	147.10 ± 4.16ab (45%)	33.79 ± 1.22abc (36%)
	T6	5.40 ± 0.31bc (244%)	31.01 ± 2.20fg (52%)	4.00 ± 3.25bc (69%)	41.08 ± 4.34c (221%)	74.56 ± 3.21de (63%)	63.50 ± 3.32ab (−0.42%)	184.67 ± 5.46a (82%)	35.52 ± 1.87ab (43%)

Average ± standard error from four separate replicates. Values with different letters are significantly determined by Duncan’s test. CK = control, T2 = Recommended dose of herbicide (2,4-D), T3 = 4 dS m^−1^; T4 = 8 ds m^−1^; T5 = 4 ds m^−1^ + Recommended dose of herbicide, and T6 = 8 dS m^−1^ + Recommended dose of herbicide. FW = Fresh weight, % = percentage, dS m^−1^ = deciSiemens per meter.

Both mild and severe saline stress treatments (T_3_ and T_4_) significantly enhanced the production of H_2_O_2_ and O_2_^−.^ in rice cultivars differentially. The sensitive cultivar exhibited 1.7 fold and 2.1 fold enhancement in H_2_O_2_ content under mild and severe saline stress treatments (T3 and T4), respectively. However, the resistant cultivar showed 1.3 fold and 1.8 fold increase in H_2_O_2_ content under the mild (T_2_) and severe (T_3_) saline stress treatments, respectively. The maximum O_2_^−.^ generation rates was recorded in the sensitive cultivar under both saline treatments (T_3_ and T_4_) as compared to the XS 134 cultivar. Under combined treatments (T_5_ and T_6_), the 2,4-D application considerably reduced H_2_O_2_ and O_2_^−.^ production in the XS 134 cultivar followed by ZJ 88. To further confirm ROS results, we performed *in situ* histochemical staining for hydrogen peroxide (H_2_O_2_) (Fig. 1C and D) and superoxide anion radicals (O_2_^−^) (Fig. 1E and F). Compared with control roots samples, 2,4-D and salt treated root were stained extensively at meristem and elongation zone with 3,3′-diaminobenzidine (DAB) (dark-brown) or nitro blue tetrazolium (NBT) (purple-blue) in sensitive cultivar (ZJ 88) compared with resistant cultivar (XS 134). However, combined stress treatments (T5 and T6) displayed relatively light staining. These results suggest that application of 2,4-D under salinity may decrease lipid peroxidation by reducing ROS (O_2_^−.^ and H_2_O_2_) formation.

### Response of enzymatic antioxidants

To investigate how 2,4-D herbicide application influence antioxidative defense system under saline stress conditions, we measured antioxidant enzyme activities such as SOD, CAT, POD and APX from both cultivars under control and stressed conditions. Salt stress and application of 2,4-D caused a significant elevation in SOD activity in both rice cultivars under different treatment regimes. However, the combined stress treatments (T5 and T6) decreased the SOD activity in the XS 134 cultivar followed by ZJ 88 (Table 2). CAT activity was increased up to 11% and 30% in the mild salt treatment (T3) in the roots of the ZJ 88 cultivar and XS 134, respectively. The application of the 2.4-D alone (T2) further enhanced the activity of CAT in both rice cultivars. At treatment T6 (severe salt stress + 2,4-D), CAT activity was significantly inhibited in cultivar ZJ 88, while it was enhanced (14%) in the roots of the XS 134 cultivar under T6 as compared to their respective salt stress treatment (T3).

The POD activity was significantly decreased in the ZJ 88 cultivar as compared to XS 134 under the herbicide treatment alone (T2). However, its activity was slightly improved under the saline (T3 and T4) and combined stress treatments (T5 and T6) in XS 134 cultivar. On the other hand, the POD activity was significantly increased in the ZJ 88 cultivar up to 21% and 32% under the mild (T3) and severe (T4) saline stress treatments. Whereas, application of herbicide further improved the POD activity in the roots of the ZJ 88 cultivar up to 28% and 49% under combined stress treatments (T5 and T6), respectively. Regarding APX activity in stress plants, the XS 134 cultivar maintained higher APX activity under mild and severe saline stress treatments as compared to the ZJ 88 cultivar. However, under the combined stress treatments (T5 and T6), no significant change was observed compared to the saline stress treatments (T3 and T4) in the ZJ 88 cultivar. Conversely, 23% and 16% increase in the APX activity was recorded for the treatment T5 and T6 for the XS 134 cultivar as compared to their respective saline stress treatments (T3 and T4).

### Effects of 2,4-D/salt on glutathione-ascorbate cycle

The glutathione reductase (GR), monodehydroascorbate reductase (MDHAR) and dehydroascorbate reductase (DHAR) are the key enzymes that are involve in the maintenance of reduced AsA and GSH contents in cell to scavenge the ROS. The mild and severe saline stress treatments (T3 and T4) significantly enhanced the GR activity in the XS 134 cultivar, while no remarkable change in the GR activity was noticed in the roots of ZJ 88 cultivar under saline treatments (T3 and T4). However, herbicide + mild salt treatment (T5) enhanced the GR activity significantly in the ZJ 88 cultivar as compared to herbicide + severe salt treatment (T6), where GR activity was increased slightly as compared to their respective salt stress treatments (T4) (Table 3). The GR activity was significantly improved under herbicide treatment alone (T2) in the XS 134 cultivar (1.9 fold) followed by the ZJ 88 cultivar (1.5 fold) compared to their respective controls. Similarly, GSH concentration was decreased, while GSSG accumulation was increased in the ZJ 88 cultivar under mild (T3) and severe (T4) saline stress conditions. However, combined treatments (T5 and T6) considerably enhanced GSSG accumulation and decreased GSSG concentration in stressed plants of the ZJ 88 cultivar as compared to their respective salt stress treatments (T3 and T4). In the resistant cultivar (XS 134), GSH accumulation increased with increasing saline stress level, while GSSG concentration was reduced considerably under saline treatments (T3 and T4) (Table 3).

**Table 3:** Effects of individual and combined stresses of 2,4-D and salinity on glutathione reductase (GR), ascorbic acid (AsA), dehydroascorbate reductase (DHAR), monodehydroascorbate reductase (MDHAR), reduced glutathione (GSH), oxidized glutathione (GSSH) and ration of GSH/GSSH in the roots of two rice cultivars.

Cultivar	Treatment	GR	ASA	DHAR	MDHAR	GSH	GSSG	GSH/	Lignin (A_280_ mg ^−1^ protein)
		(U mg^−1^ protein)	(µg g^−1^ FW)	(U mg^−1^ protein)	(U mg^−1^ protein)	(nmol g^−1^ FW)	(nmol g^−1^ FW)	GSSG ratio	
ZJ 88	Control (CK)	2.11 ± 0.16 f (100%)	35.82 ± 2.36d (100%)	11.21 ± 1.07bc (100%)	3.80 ± 0.40c (100%)	0.42 ± 0.02 cd (100%)	0.117 ± 0.01bc (100%)	3.68 ± 0.34ef (100%)	7.43 ± 0.66 (100%)
	T2	3.34 ± 0.39ef (58%)	39.50 ± 1.49bcd (10%)	12.60 ± 1.71abc (12%)	6.77 ± 0.69ab (78%)	0.49 ± 0.03abcd (17%)	0.107 ± 0.01bc (−9%)	4.61 ± 0.15bc (25%)	8.05 ± 0.58 (8%)
	T3	3.04 ± 0.30ef (44%)	38.12 ± 2.94 cd (6%)	13.16 ± 1.13abc (17%)	3.52 ± 0.55c (−7%)	0.42 ± 0.01 cd (0%)	0.137 ± 0.01ab (17%)	3.15 ± 0.32fg (−14%)	11.40 ± 0.87 (53%)
	T4	3.09 ± 0.42ef (46%)	36.23 ± 3.88d (1%)	15.82 ± 1.42a (41%)	3.25 ± 0.58c (−14%)	0.44 ± 0.02bcd (5%)	0.137 ± 0.02ab (17%)	3.24 ± 0.16fg (−12%)	11.86 ± 0.96 (60%)
	T5	5.85 ± 0.52 cd (177%)	42.86 ± 1.24ab (20%)	12.97 ± 1.00abc (16%)	5.22 ± 0.65bc (37%)	0.50 ± 0.02abcd (19%)	0.117 ± 0.01bc (0%)	4.31 ± 0.27 cd (17%)	14.20 ± 1.64 (91%)
	T6	4.22 ± 0.34de (100%)	39.15 ± 1.60bcd (9%)	12.78 ± 0.92abc (14%)	8.19 ± 1.03a (116%)	0.51 ± 0.04abc (21%)	0.123 ± 0.01abc (5%)	4.17 ± 0.41 cd (13%)	14.64 ± 1.80 (97%)
XS 134	Control (CK)	3.25 ± 0.20ef (100%)	36.49 ± 2.14d (100%)	10.66 ± 0.75c (100%)	3.59 ± 0.26c (100%)	0.40 ± 0.03d (100%)	0.150 ± 0.02a (100%)	2.68 ± 0.09 g (100%)	8.10 ± 0.83 (100%)
	T2	6.02 ± 0.77 cd (85%)	39.82 ± 1.17bcd (9%)	12.76 ± 0.75abc (20%)	6.93 ± 0.31ab (93%)	0.47 ± 0.02bcde (18%)	0.113 ± 0.01bc (−24%)	4.15 ± 0.23 cd (55%)	10.23 ± 1.13 (26%)
	T3	6.56 ± 0.55bc (102%)	40.71 ± 1.63abc (12%)	13.79 ± 0.67abc (29%)	4.09 ± 0.72c (14%)	0.47 ± 0.02bcde (18%)	0.120 ± 0.01abc (−20%)	3.93 ± 0.16def (47%)	10.63 ± 1.04 (31%)
	T4	8.15 ± 0.81ab (151%)	43.72 ± 1.21ab (20%)	14.38 ± 1.20ab (35%)	4.40 ± 0.57c (23%)	0.52 ± 0.03abc (30%)	0.100 ± 0.01c (−33%)	5.20 ± 0.42ab (94%)	12.95 ± 0.89 (60%)
	T5	8.67 ± 0.33b (167%)	45.49 ± 3.58a (25%)	14.93 ± 1.31a (40%)	6.53 ± 0.23ab (82%)	0.55 ± 0.01a (38%)	0.097 ± 0.001c (−35%)	5.70 ± 0.11a (113%)	17.67 ± 1.21 (118%)
	T6	10.48 ± 1.28a (223%)	45.83 ± 2.64a (26%)	15.47 ± 0.85a (45%)	8.95 ± 0.87a (149%)	0.54 ± 0.02ab (35%)	0.093 ± 0.001c (−38%)	5.80 ± 0.34a (116%)	19.30 ± 1.04 (138%)

Average ± standard error from four separate replicates. Values with different letters are significantly different at *P* ≤ *0.05* as determined by Duncan’s test. CK = control, T2 = Recommended dose of herbicide (2,4-D), T3 = 4 dS m^−1^, T4 = 8 dSm^−1^, T5 = 4 ds m^−1^ + Recommended dose of herbicide, and T6 = 8 dS m^−1^ + Recommended dose of herbicide. FW = Fresh weight, dS m^−1^ = deciSiemens per meter.

The activity of DHAR was increased in both cultivars under saline stress, however, no further increase in DHAR activity was found in the ZJ 88 cultivar as compared to their respective salt stress treatments (T3 and T4) under combined treatments (T5 and T6). However, the XS 134 cultivar maintained a higher DHAR activity level under both saline stress (T3 and T4) and combined stress treatments (T5 and T6). The saline stress treatments (T3 and T4) significantly enhanced the accumulation of AsA in the XS 134 cultivar, while in the sensitive cultivar AsA content was considerably increased under mild saline stress treatment, while no change in AsA concentration was found under severe saline stress treatment as compared to the control plants. Although, decrease of AsA in the sensitive cultivar was restored under mild saline stress + 2,4-D treatment (T5) as compared to the severe saline stress + 2,4-D treatment (T6), where AsA was increased non-significantly (Table 3). MDHAR activity was decreased as compared to the control level under both saline stress treatments (T3 and T4) in the ZJ 88 cultivar, while no significant change in MDHAR activity was detected under saline stress treatments in the XS 134 cultivar as compared to the control plants. The addition of 2,4-D enhanced the activity of MDHAR in all treatments in both cultivar except in the ZJ 88 cultivar at treatment T6. The XS 134 cultivar showed greater ability to tolerate saline stress, which may be associated with modulated activities of the GR, DHAR and higher accumulation of GSH and AsA under stress conditions as compared to the ZJ 88 cultivar. These results indicate that superior maintenance of enzymes of glutathione-ascorbate cycle might play critical role in the plants improved tolerance under combined treatments as compared to the individual stress treatments.

### Effects of 2,4-D/salt on gene expression of antioxidant defense enzymes

**Figure 2:**
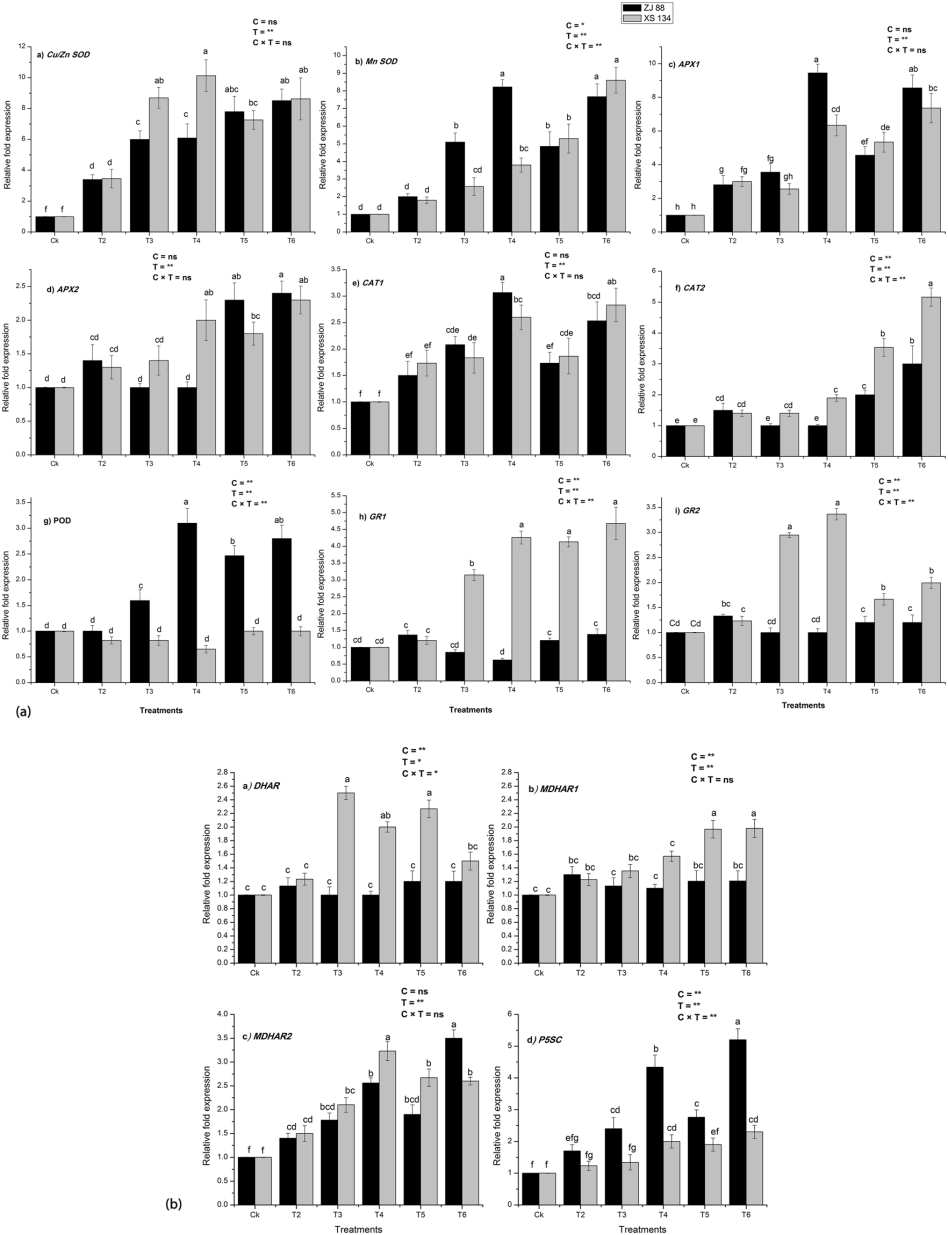
(**a**) Relative fold expression of the genes encoding antioxidant defense enzymes. (a) *Cu/Zn SOD*, (b) *Mn SOD*, (c) *APX1*, (d) *APX2*, (e) *CAT1*, (f) *CAT2*, (g) *POD*, (h) *GR2*, (i) *GR1*, (j) *DHAR*, (k) *MDHAR1*, (l) *MDHAR2*, (m) *P5SC* in the roots of two rice cultivars (ZJ 88 and XS 134) grown under herbicide and saline stress conditions. Average ± standard error from three separate replicates. 20-days-old rice seedlings were treated as control (CK) with EC 1.2 dS m^−1^, T2 (recommended dose of 2,4-D), T3 (EC 4 dS m^−1^), T4 (EC 8 dS m^−1^), T5 (EC 4 dS m^−1^ + recommended dose of 2,4-D), T6 (EC 8 dS m^−1^ + recommended dose of 2,4-D) for 15 days. Afterwards, samples were collected for expression analysis. **(b**) Relative fold expression of the genes encoding antioxidant defense enzymes. (a) *DHAR*, (b) *MDHAR1*, (c) *MDHAR2*, d) *P5SC* in the roots of two rice cultivars (ZJ 88 and XS 134) grown under herbicide and saline stress conditions. Average ± standard error from three separate replicates. 20-days-old rice seedlings were treated as control (CK) with EC 1.2 dS m^−1^, T2 (recommended dose of 2,4-D), T3 (EC 4 dS m^−1^), T4 (EC 8 dS m^−1^), T5 (EC 4 dS m^−1^ + recommended dose of 2,4-D), T6 (EC 8 dS m^−1^ + recommended dose of 2,4-D) for 15 days. Afterwards, samples were collected for expression analysis.

To better understand the enhanced salt tolerance mechanism exhibits by the rice cultivars under combined and individual stress treatments, the transcript level of antioxidant enzymes was analyzed through quantitative qRT-PCR. The expression levels of the SODs isozymes were relatively higher in the ZJ 88 cultivar as compared to the XS 134 under severe saline (T4) as compared to mild saline stress treatment (T3), respectively. Transcript level of the *cytosolic SOD* under combined treatments was reduced in the XS 134 cultivar as compared to the ZJ 88. Conversely, *Mn SOD* expression level was enhanced in the ZJ 88 cultivar as compared to the XS 134 under saline stress treatments. However, the addition of the 2,4-D alone considerably modulated the transcript level of the *SOD* isozymes in the root of rice cultivars (Fig. 2a). The transcript levels of two *cytosolic APX* genes showed considerable induction under 2,4-D + severe saline stress treatment (T6) followed by 2,4-D + mild saline stress treatment (T5). Under saline stress treatments (T3 and T4), the *APX*2 transcript level was not changed in the ZJ 88 cultivar compared to their respective control (Fig. 2a).

Transcript abundance of the peroxisomes catalase (*CAT1*) gene was considerably enhanced under mild and severe saline stress condition in both cultivars. While, the *CAT1* gene expression was reduced in the ZJ 88 cultivar under combined treatments, whereas no considerable induction was observed in the XS 134 cultivar compared to their respective saline stress treatments (Fig. 2a). In addition, the ZJ 88 cultivar showed no remarkable change in the *CAT1* transcript level under saline stress treatments. The 2,4-D application under mild and severe saline stress conditions considerably induced cytosolic catalase (*CAT2*) expression in the ZJ 88 cultivar as compared to saline stress treatments (T3 and T4). The expression level of the *CAT2* in the XS 134 cultivar was remarkably enhanced under saline (T3 and T4) and combined treatments (T5 and T6), respectively. In the XS 134 cultivar, the *POD* transcript expression was downregulated under saline stress conditions, while the ZJ 88 maintained a considerable higher transcript level under mild (1.5 fold) and severe (3.1 fold) saline stress treatment (Fig. 2a). The combined treatments further enhanced the transcript abundance of the *POD* in the ZJ 88 cultivar, while it’s expression was not improved in the XS 134 cultivar as compared to control. A differential expression pattern of the two GR isoforms were observed in rice cultivars under stress conditions. The cytosolic *GR* (*GR1*) expression was downregulated, whereas mitochondrial *GR* (*GR2*) expression was stable in the ZJ 88 cultivar under saline stress treatments (T3 and T4). While, *GR1* and *GR2* expression levels were remarkably upregulated in the XS 134 cultivar by salt stress under T3 and T4 treatment, respectively. The addition of 2,4-D enhanced the *GR1* and *GR2* expression more in the XS 134 cultivar as compared to the ZJ 88 cultivar under combined stress treatments (Fig. 2b).

In the ZJ 88 cultivar, DHAR transcript level was not changed considerably under individual and combined stress conditions. In case of the XS 134 cultivar, significant increase in the DHAR transcript accumulation was observed under saline stress treatments (T3 and T4), while under combined stress treatments, DHAR transcript accumulation was lower than saline stress treatments (Fig. 2b). The 2,4-D treatment also improved the *DHAR* transcript accumulation more in the resistant cultivar as compared to the sensitive cultivar (Fig. 2b). The transcript accumulation of both *cytosolic MDHAR 1* and *2* genes were relatively higher in the XS 134 cultivar as compared to the ZJ 88 cultivar. Under saline stress treatments, cultivar XS 134 expressed higher transcript expression of *MDHAR* isoforms compared to the sensitive cultivar. Combined treatments also increased the transcript abundance considerably in cultivar XS 134 as compared to cultivar ZJ 88 (Fig. 2b). In the present investigation, gene expression patterns at the mRNA level and changes in enzyme activity under different stress treatments, revealed no positive correlation under some treatments, which might be explained by the regulation of genes at the post-transcriptional, translational, or post-translational levels. These results suggest that the up-regulated gene expressions were likely to be involved in enhancing the activities of antioxidant enzymes in cultivar XS 134 and subsequently increase stress tolerance. On the basis of these results, we speculate that salt/2.4-D application alone and their combination differently modulated the antioxidant defense system of the resistant and the sensitive cultivar.

### Gene expression of Na^+^ and K^+^ transporters genes

**Figure 3:**
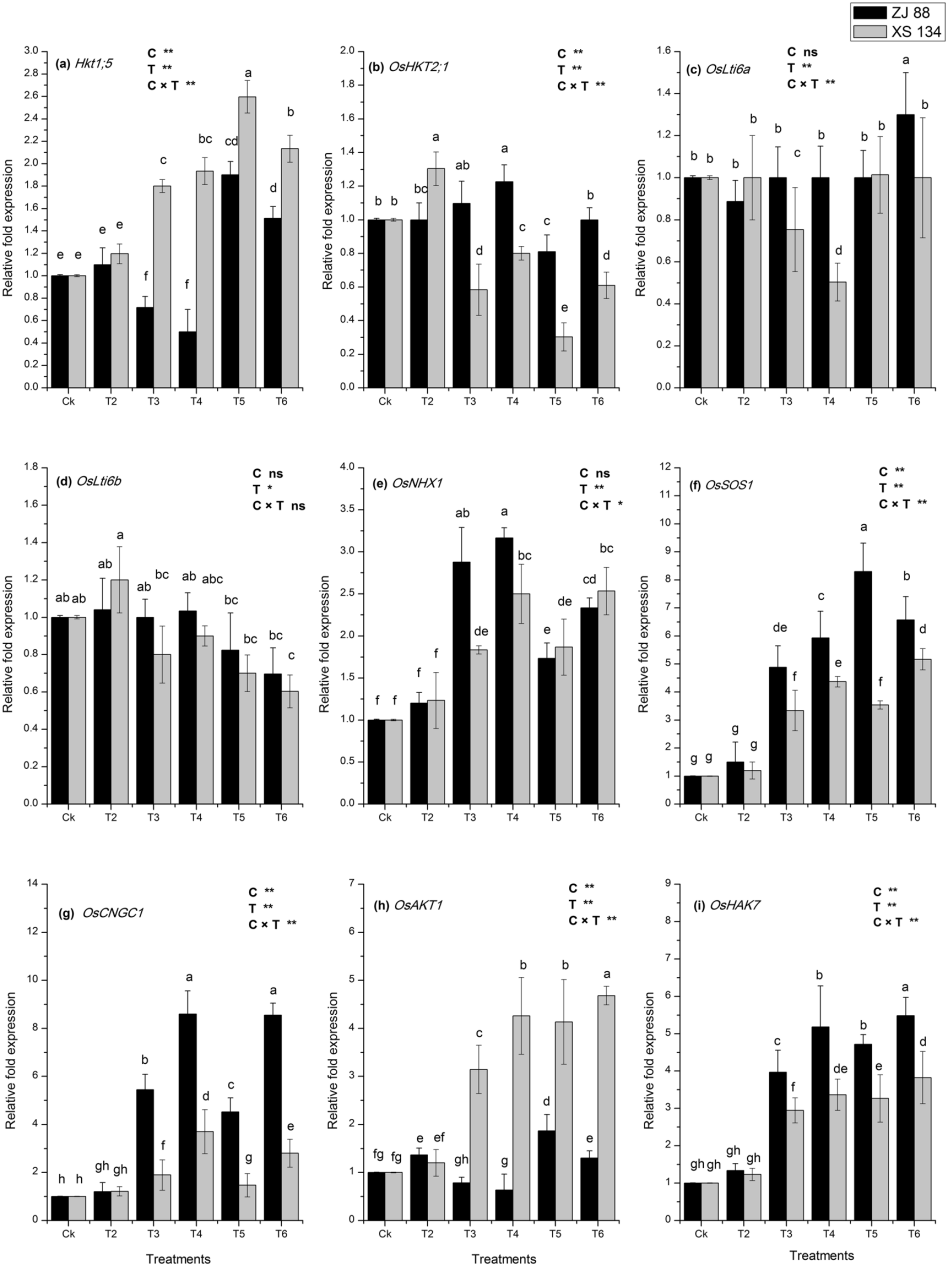
Relative fold expression of the genes encoding Na^+^ and K^+^ transporter proteins (**a**) *OsHKT1;5*, (**b**) *OsHKT2;1*, (**c**) *OsLti6a*, (**d**) *OsLti6b*, (**e**) *OsSOS1*, (**f**) *OsCNGC1*, (**g**) *OsAKT1* (**h**) *OsAKT7* (**i**) *OsNHX1* in the roots of two rice cultivars (ZJ 88 and XS 134) grown under herbicide and saline stress conditions. Average ± standard error from three separate replicates. 20-days-old rice seedlings were treated as control (CK) with EC 1.2 dS m^−1^, T2 (recommended dose of 2,4-D), T3 (EC 4 dS m^−1^), T4 (EC 8 dS m^−1^), T5 (EC 4 dS m^−1^ + recommended dose of 2,4-D), T6 (EC 8 dS m^−1^ + recommended dose of 2,4-D) for 15 days. Afterwards, samples were collected for expression analysis.

The expression profiles of the genes encoding Na^+^ and K^+^ transporter proteins were analyzed in two contrasting rice cultivars roots to determine the underlying mechanisms of differential Na^+^ and K^+^ accumulation under different stress conditions (Fig. 3). A *OsHKT1;5* is a Na^+^ transporter, located in the root xylem parenchyma to efflux Na^+^ form xylem sap to prevent its translocation to the shoot. Our results showed that *OsHKT1;5* expression was downregulated under mild (T3) and severe (T4) saline stress conditions in the ZJ 88 cultivar, while its expression was considerably increased in the XS 134 cultivar under both saline stress treatments (T3 and T4). The combined treatment of 2,4-D under mild and sever saline stress considerably enhanced the expression of *OsHKT1;5* in both cultivars roots. However, its expression was more pronounced in the XS 134 cultivar as compared to the ZJ 88.

The expression of another Na^+^ transporter protein *OsHKT2;1* was enhanced up to 0.10 and 0.20 fold respectively in the ZJ 88 cultivar, while 0.40 and 0.20 fold decrease in the cultivar XS 134 respectively under mild (T3) and severe (T4) saline stress conditions. Similarly, the expression pattern of *OsHKT2;1* under combined treatments (T5 and T6) was further downregulated in both cultivar as compared to control. *OsLti6a* and *OsLti6b* are small size proteins in rice required for the prevention of excess Na^+^ entry in the cell. In this study, expression of *OsLti6a* in the ZJ 88 cultivar was unchanged, while it was decreased under mild (0.25 fold) and severe (0.50 fold) saline stress treatments in cultivar XS 134, respectively. However, under combined treatments (T5 and T6), *OsLti6a* expression was not changed in both cultivars except treatment T6 in the ZJ 88 cultivar, where *OsLti6a* expression was enhanced up to 0.30 fold as compared to control. The combined treatment T6 remarkably reduced the expression of *OsLti6b* in both cultivars; however *OsLti6b* expression did not change in the ZJ 88 cultivar under saline stress conditions (T3 and T4) compared with control (Fig. 3).

The *OsNHX1* is a vacuolar Na^+^, K^+^/H^+^ antiporter, sequester surplus Na^+^ from cytosol into the vacuole under saline stress conditions. A remarkable enhancement in the transcript expression of *OsNHX1* was recorded in rice cultivar roots under different treatments. Mild (T3) and severe (T4) saline stress treatment considerably induced its expression up to 2.9 and 3.2 fold in the ZJ 88 cultivar, whereas in the XS 134 cultivar less induction in transcript abundance of *OsNHX1* was observed as compared to the ZJ 88 cultivar. However, combined treatments (T5 and T6) downregulated the *OsNHX1* expression in roots of ZJ 88, while no considerable change in transcript expression was observed in the XS 134 cultivar as compared to mild (T3) and severe (T4) saline stress treatments, respectively. The transcript expression of another Na^+^/H^+^ antiporter salt overly sensitive 1 (*SOS1*) is involved in regulation of Na^+^ from cytosol was considerably induced in both rice cultivars under saline stress conditions (T3 and T4) (Fig. 3). The addition of 2,4-D under mild (T3) and sever (T4) saline stress conditions further enhanced the transcript abundance of *OsSOS1* in the ZJ 88 cultivar as compared to XS 134. Cyclic nucleotide-gated channels (CNGCs) are non-selective cation transporters like *AtCNGC1* mainly function in the uptake of Na^+^ into root cells under saline stress conditions. Our result revealed that the expression of *AtCNGC1* was much higher in the roots of ZJ 88 cultivar under mild (5.5 fold) and severe (8.6 fold) stress conditions as compared to XS 134 cultivar. Conversely, its expression was reduced under combined stress conditions (T5 and T6) in both cultivars except treatment T6 in ZJ 88 cultivar, where the expression of *AtCNGC1* was not changed compared to its respective salt stress treatment (T3) (Fig. 3).

Potassium inward rectifying channels, such as *OsAKT1* have high K^+^/Na^+^ selectivity and mediate active K^+^ influx under salinity. Under saline stress conditions, *OsAKT1* was downregulated in the cultivar sensitive (ZJ 88), while its transcript expression was considerably upregulated in the resistant cultivar (XS 134) (Fig. 3). Addition of 2,4-D under saline stress condition considerably enhanced *OsAKT1* expression in both cultivars, but in the resistant cultivar its expression level was much higher as compared to the sensitive cultivar (ZJ 88). Another potassium transporter *OsHAK7* expression was considerably increased in cultivar ZJ 88 under all stress treatments as compared to the resistant cultivar (XS 134). The qRT PCR results of the present study about Na^+^, K^+^ transports and vacuolar compartmentalization proteins showed differential activation patterns that could played an essential role in K^+^ entry and Na^+^ efflux/efflux in the roots of the resistant and sensitive rice cultivars under saline (T3 and T4) and combined stress (T5 and T6) treatments (Fig. 3).

### Callose and lignification of roots under individual and combined stress treatments

**Figure 4:**
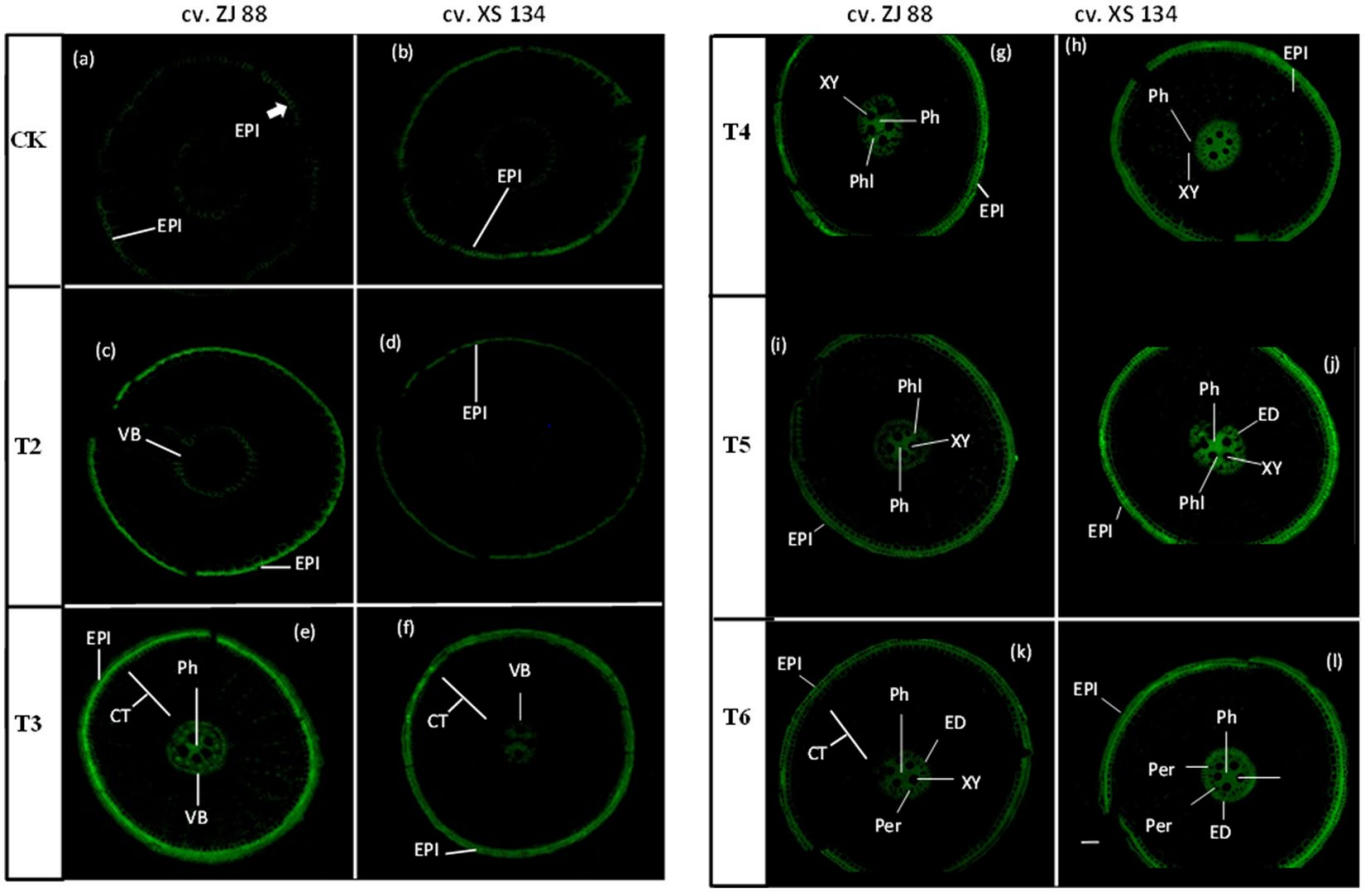
Callose deposition in roots of rice cultivars (ZJ 88 and XS 134). Rice cultivars seeding were exposed to 2.4-D, NaCl and combined stress treatments for 15 days. Afterwards, Root tips (0.5 cm) were fixed with 85% ethanol, stained with 1% aniline blue in 1 M glycine (pH 9.5), then cross-sectioned and examined under a fluorescence microscopy (NIKON ELIPSE N*i* equipped with LEICA DFC 425 camera). A slight development of callose was observed at epidermal region in cultivar ZJ 88 followed by cultivar XS 134 under control (**a**,**b**) and 2,4-D alone treatments (T1) (**c**,**d**). Lower (**e**,**f**) and higher (**g**,**h**) saline stress treatments (T3 and T4) showed more development of callose in dose depended manner. Staining of combined stress treatments (T5 and T6) showed that more development of callose in cultivar XS 134 (**i**,**k**) as compared to the cultivar ZJ 88 (**j**,**l**). 20-days-old rice seedlings were treated as control (CK) with EC 1.2 dS m^−1^, T2 (recommended dose of 2,4-D), T3 (EC 4 dS m^−1^), T4 (EC 8 dS m^−1^), T5 (EC 4 dS m^−1^ + recommended dose of 2,4-D), T6 (EC 8 dS m^−1^ + recommended dose of 2,4-D) for 15 days. CT = cortex; EPI = epidermis; VB = vascular bundles; Ph = pith; Phl = phelom; XY = xylem; ED = endodermis; and Per = pericycle. Scale bar = 0.2 mm.

The effects of salt stress and herbicide application on rice cultivars roots were further examined by visualizing callose, which is a vital component of plant cell wall. Exposure of rice cultivars under herbicide treatment alone did not result in distinct callose fluorescence in the root; however, most of the cross-sections exhibited minor fluorescence compared with control. Saline stress enhanced the production of callose under lower and higher saline stress treatments (Fig. 4) in a dose dependent manner. The fluorescent signal was increased at both epidermal region and in the central vascular cylinder in cultivar XS 134 compared with cultivar ZJ 88, under saline stress treatments (Fig. 4e–h). Under combined stress (T5 and T6), cultivar XS 134 showed increase fluorescence in the central vascular cylinder and at the epidermal region as compared to the cultivar ZJ 88 (Fig. 4i,k,l). But, fluorescence under combined treatments was stronger as compared with saline stress treatments (T2 and T3).

Total lignin content in roots was measured by the photometric method. The roots grown in saline stress had a significantly higher amount of lignin, both under mild and severe saline stress conditions, than those of control roots. Cultivar ZJ 88 showed significant increase in lignin production under severe saline stress treatment compared to XS 134, where lignin accumulation was increased considerably compared to T3 treatment (Table 3). 2,4-D application alone (T2) did not alter the lignin production in roots of rice cultivars. However under combined stress treatments, 1.8 fold and 2 fold increase in lignin production was recorded in the cultivar ZJ88 and XS 134 under treatment T5, respectively. Conversely, further increase in lignin production was restricted in the ZJ 88 cultivar (Table 3), while XS 134 enhanced lignin production up to 2.4 fold under combined treatment T6 compared to control. This shows that combined stress treatments differentially impacted the lignification process in roots of rice cultivars.

**Figure 5:**
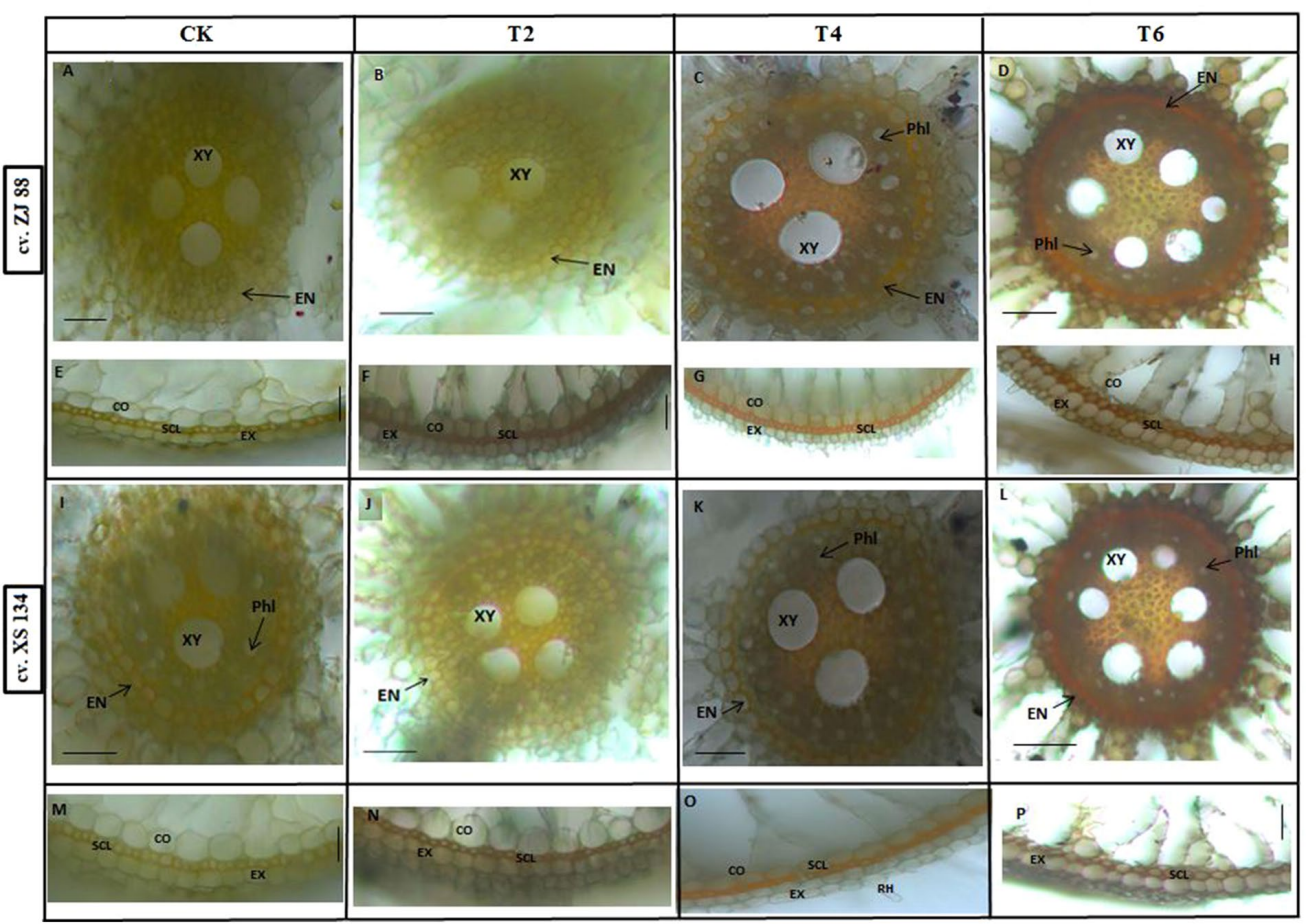
Comparison of lignin deposition in rice roots. Lignin staining in the central cylinder (CC) (**A**–**D** and **I** to **L**) and the outer part of the roots (**E**–**H** and **M**–**P**) grown in salt and 2,4-D treatments. Lignin in the cell walls was detected by an orange/brown staining. Root tips (0.5 cm) were stained with Maule reaction, then cross-sectioned and examined under a light microscopy (LEICA MZ 95 microscope equipped with LECIA DFC 300 FX camera). A slight development of lignin was observed in the central cylinder (CC) of roots under 2,4-D treatments (**B**,**J**) in both rice cultivars. Relatively brighter staining in the CC of roots of cultivar ZJ 88 (**C**,**G**) compared to XS 134 (**K** and **O**) was found under higher saline stress treatment (T4). Under higher saline and 2,4-D combined treatment (T6), staining turned to reddish brown, showing intense lignin deposition in endodermis cell and its adjacent cell layers in cultivar XS 134 followed by ZJ 88. Under 2,4-D alone treatment (**F**,**N**) thin, light brown sclerenchyma and exodermal cell walls in outer part of the roots (OPR) of rice cultivars can be found. Under higher saline stress treatment, sclerenchyma cells with faint orange color can be observed in the figures (**G**,**O**). Intense reddish brown stains in root of the cultivar XS 134 followed by cultivar ZJ 88 can be seen under combined salt and herbicide stress treatment (**H**,**P**). 20-days-old rice seedlings were treated as control (CK) with EC 1.2 dS m^−1^, T2 (recommended dose of 2,4-D), T4 (EC 8 dS m^−1^), T6 (EC 8 dS m^−1^ + recommended dose of 2,4-D) for 15 days. Scale bar = 0.05 mm.

Lignin was also detected by an orange/brown staining of roots cross sections using the Maüle reaction (Fig. 5). When rice cultivars roots were exposed to the herbicide application alone, slight development of lignin was observed in the central cylinder (CC) including endodermis cells and sclerenchyma cells of the outer part of the root (OPR) (Fig. 5B,J,F and N). In contrast, results demonstrated that roots from higher salt stress developed higher lignification of whole stele, including the endodermis; however the ZJ 88 cultivar had more intense sainting than the XS 134 cultivar. Whereas, roots from lower saline stress treatment in both rice cultivars showed mild staining intensity (data not shown). However, under higher saline + herbicide treatment (T6), sclerenchyma cells and exodermal cells in OPR had the most intense staining with compact and thick walls in the XS 134 cultivar as compared to the ZJ 88 cultivar. Similarly, endodermis cells and its adjacent cell layers at central cylinder (CC) were also positively stained by Maüle reagent with higher relative staining intensity in the XS 134 cultivar compared to the ZJ 88.

### Root ultra-structure under individual and combined stress treatments

**Figure 6:**
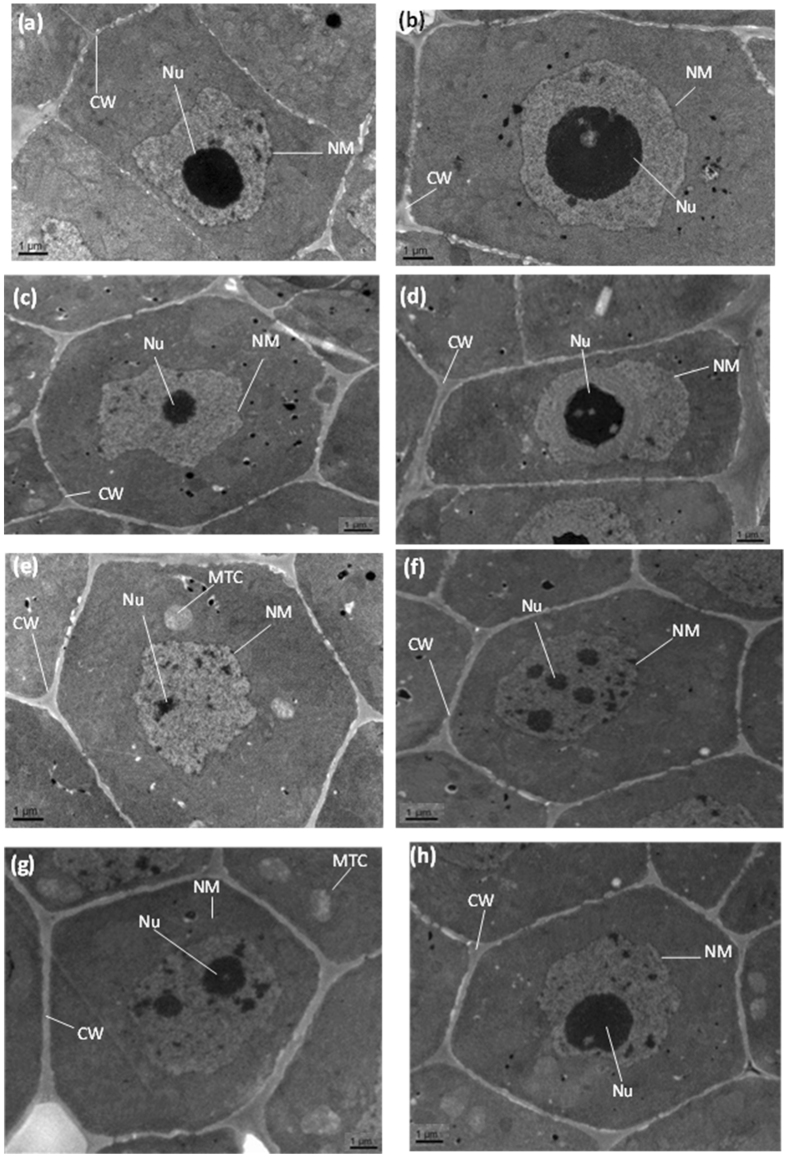
Electron micrographs of root tips of 15 days-treated rice cultivars (cvs. ZJ 88, XS 134) under control (CK), 2,4-D (T2), sever saline stress (T4) and combined stress treatments (T6). Root tip cells of ZJ 88 and ZX 134 under control (CK) shows well organized internal structure of root cell with smooth nuclear membrane (NM), well developed nucleolus and nucleus (Nu) (**a,b**). TEM micrographs of root tip cells of ZJ 88 and XS 134 show condense nucleolus and disrupted nucleus (Nu) but less nuclear membrane damage (**c,d**). TEM micrographs of root tip cells of ZJ 88 and XS 134 also show abnormal mitochondria structure (MTC), rupture nuclear membrane (NM) disintegrated nucleolus (**e,f**). Root tip cells of ZJ 88 and XS 134 show relatively greater size of nucleolus with swollen mitochondria (MTC) and thick cell walls (**g** and **h**). 20-days-old rice seedlings were treated as control (CK) with EC 1.2 dS m−1, T2 (recommended dose of 2,4-D), T4 (EC 8 dS m−1), T6 (EC 8 dS m−1 + recommended dose of 2,4-D) for 15 days. Scale bars = 1 μm. The experiment was repeated three times with similar results.

Transmission electron microscopy of root tips of rice cultivars under control conditions showed well-structured cytoplasmic matrix with smooth and continuous cell walls and bigger nucleus and nucleolus relative to the plants exposed to stress conditions (Fig. 6a and b). In contrast, roots exposed to the 2,4-D/salt stress alone or combined, numerous alterations were noticed in the ultra-structure of root cells. Under 2,4-D treatment alone (T2), condensation of nucleolus, disruption of nucleus and nuclear membrane were found in both rice cultivars roots (Fig. 6c and d). However, under severe saline treatment (T4), swelling of mitochondria, distorted cristae, disruption of nucleus, disappearance of nucleolus, and disruption of nuclear membrane can be seen in the ZJ 88 cultivar followed by the XS 134 cultivar cells (Fig. 6e and f). Conversely, damage caused by combined treatment (T6) to the nucleus and other organelles was less in comparison with plants exposed to 2,4-D or NaCl stress alone in the XS 134 cultivar compared to the ZJ 88 (Fig. 6g and h).

## Discussion

An understanding of salt tolerance mechanisms is essential for the development of salt resistant cultivars to cope with growing salinized agricultural land^8–10^. Rice is considered a staple food source and its relatively higher sensitivity to salinity makes it necessary to improve rice salt tolerance, but doing so requires a detailed understanding of the mechanism, genes and proteins involved in the tolerance/resistance process. A comparison of the physiological, biochemical and molecular characteristics of cultivars with contrasting levels of sensitivity/resistance can provide detailed insights into the underlying mechanism^32, 33^. For decades, auxinic herbicides (like 2,4-D) have been used to control broadleaf plants, and have played a critical role in weed control^16, 19^. The 2,4-D herbicide is a mimicker of natural auxin and acts like a plant hormone (auxin) at the molecular level^19^. Due to its widespread application in agriculture, including on paddy fields^16, 22^, it is worthwhile to investigate the effects of 2,4-D application under abiotic stress conditions. Therefore, in this investigation, we examined the effects of the recommended dose of 2,4-D on the growth of rice roots under saline stress conditions. We focus on roots because, most of the studies have been performed on leaves and little information is available about roots, even though it is the first organ directly exposed to the salt/herbicides.

Environmental stresses such as salinity and herbicides lead to the disruption of the balance between ROS production and scavenging, resulting in oxidative bursts^24, 34–38^. ROS production in saline-stressed roots can be produced by mitochondria, peroxisomes or by apoplastic cell wall peroxidases, amine oxidases and plasma membrane nicotinamide adenine dinucleotide phosphate-oxidase^39^. However, an increasing amount of evidence suggests that auxin herbicides can also produce ROS (O_2_^−^, H_2_O_2_) in susceptible plants through the activation of xanthine oxidase, acyl-CoA oxidase, and NADPH oxidases enzymes^25, 40^. In this study, individual saline and herbicide treated plants showed strong DAB and NBT staining for H_2_O_2_ and O_2_^−^ in roots compared with combined stress treatments (Fig. 1). Our results further suggest that roots of the sensitive cultivar ZJ 88 were more sensitive to saline and herbicide treatments than were those XS 134 in terms of the *in situ* accumulation of H_2_O_2_ and O_2_^−^. Consistently, a spectroscopic analysis of H_2_O_2_ and O_2_^−^ further confirmed the results of DAB and NBT staining (Fig. 1). Excessive ROS production during stress could result in serious damage to the cytomembrane and to the vital cellular macromolecule^25, 39^. Lipid peroxidation and electrolyte leakage are considered effective indicators of cellular oxidative damage^41^. Thus, less damage from membrane lipid peroxidation and EL enabled the XS 134 cultivar to continue normal metabolism under stress conditions, contributing to its higher salt tolerance^8, 9^.

To mitigate oxidative damage under adverse conditions, plants have developed an antioxidant defense system that includes enzymatic and non-enzymatic antioxidants^42, 43^. In this study, a positive correlation between enzymatic activity of SOD and ROS (H_2_O_2_, O^−^_2_) accumulation was noted for cultivar XS 134 (r = 0.94, P < 0.05) and ZJ 88 (r = 0.91, P < 0.05) under individual and combined stress conditions (Table 2). Under saline/pesticide stress, higher transcript levels of the genes coding SOD enzymes were observed by different authors^44–47^. Hernandez, *et al*.^48^ reported higher transcript levels of *MnSOD* and *CuZnSOD* in maize and tomato under saline/drought stresses and argued that they were involved in saline induced oxidative stress tolerance^47, 49^. In *Bruguiera gymnorrhiza, Nicotiana tabacum* and *Oryza sativa* increased transcript level of cytosolic *CuZn SOD/Mn SOD* was found to enhance salt tolerance^50–52^. Manganese SOD (*Mn-SOD*) is a key enzyme that functions in controlling the ROS signaling pathway^47^. In this study, the higher transcript level of *Cu/Zn SOD* in cultivar XS 134 versus salt sensitive cultivar suggests that activation of this stress responsive gene could be involved in the antioxidant protection of rice cultivars during stress conditions^44, 46, 53^.

A decrease in ROS accumulation in salt treated roots was associated with an increase or decrease of antioxidant enzymes activities in the resistant cultivar (XS 134). However, in the sensitive cultivar, the higher accumulation of ROS might be due to a less induction of CAT enzymatic activity compared with resistant cultivar XS 134 (Table 2). Previous studies have suggested that CAT has less affinity for H_2_O_2_, but that its activity is indispensable under higher levels of ROS production, whereas APX is involved in the fine modulation of ROS in signaling despite its higher affinity for H_2_O_2_^54, 55^. Generally, the transcript accumulation of cytosolic CAT gene (*CAT 1*) was higher in sensitive cultivar under individual stress conditions, while peroxisome CAT gene (*CAT 2*) was strongly expressed in resistant cultivar under saline stress conditions. The diverse effects of the individual and combined stress conditions on mRNA abundance of CAT genes provide an insight into the mode of action of different stress treatments and their effect on maintenance of redox balance^44, 56^. The overexpression of APX in enhanced stress tolerance has been well documented by several authors^57–60^. The activity of APX was higher in the resistant cultivar as compared to the sensitive cultivar under herbicide alone/combined stress treatments. In rice, the cytosolic APX genes were up regulated under different stress conditions, including salinity suggesting their protective role under stressful conditions^61^. Previously, Rosa *et al*.^62^ reported that the silencing of only one gene encoding cytosolic APX can impair the normal development and functioning of the antioxidative system, thus making the plants more vulnerable to stress. The strong stimulation of the cytosolic APX gene expression in response to individual and combined stress conditions suggests that cytosolic APX activity is fundamental to scavenging the excess of H_2_O_2_ induced by the different stresses in rice plants^63^. In cultivar ZJ 88 the enhanced transcription and enzyme activity of POD may be the best strategy for coping with H_2_O_2_ and improving lignin synthesis^64^ (Table 2, Fig. 2).

In the present study, the application of 2,4 D enhanced the activation of antioxidant enzymes under saline stress conditions, which is in line with previous studies in which natural auxin and synthetic auxin (2,4-D) stimulated the activities of antioxidant enzymes, such as SOD, CAT, and APX in wheat, maize and other plants under abiotic stress conditions^65–67^. This may suggest that enhanced antioxidant capacity under combined stress conditions may be due to 2,4-D modification in the transcription of the genes that encode antioxidant enzymes. However, under stress conditions, stress-mediated changes in gene expression were not always correlated with corresponding changes in gene expression or enzyme activity^48, 68^. This discrepancy may be a result of higher turnover of these enzymes and/or an increase in their inactivation by H_2_O_2_^69^.

GSH and AsA are directly oxidized by ROS, or indirectly oxidized via coupling reactions of the ascorbate–glutathione recycling pathway^67, 70^. In this study, the GR activity significantly increased in the resistant cultivar compared to the sensitive one under saline stress and combined treatments, which correlates with the a higher accumulation of GSH in XS 134 cultivar compared with the ZJ 88 cultivar under the saline stress and combined treatments (Table 3). Different authors have suggested that higher concentration of GSH stimulates AsA regeneration via the ascorbate−glutathione cycle under stress conditions^10, 67^. This assumption is supported by the fact that the AsA accumulation was enhanced in cultivar XS 134 under saline and combined stress treatments compared with sensitive cultivar.

In the present study, a significant increase in MDHAR and DHAR activities in the resistant cultivar under both saline and combined treatments improved the AsA recycling and AsA content^71^. The MDHAR activity improved under combined treatments compared to individual treatments (Table 3). However, an expression analysis of DHAR did not change considerably under combined treatments in the sensitive cultivar, which might explain the poor redox maintenance in the ZJ 88 cultivar (Fig. 2). The application of 2,4-D stimulated the activities of enzymatic components of glutathione-ascorbate cycle in our study, which was consistent with a previous study, that showed that 2,4-D enhanced the activities of AsA-GSH cycle enzymes^24^. The transcript analysis of cytosolic (*GR1*) and mitochondrial GR (*GR2*) were strongly induced in the resistant cultivar under saline or herbicide treatments as compared to the sensitive cultivar (Fig. 2). Previously, Hong *et al*.^72^ demonstrated the induction of *GR1* and *GR2* expression in roots of rice plants under salinity. However, the application of 2,4-D under saline stress conditions strongly enhanced the expression of these GR genes which is in agreement with earlier studies on drought, salinity, chilling, cadmium, powdery mildew and ABA application^73–77^. Furthermore, Wu *et al*.^78^ proposed that cytosolic and mitochondrial GR protein responses under environmental stimuli and phytohormones in rice are diverse. Together, these results indicate that the application of 2,4-D can regulate AsA regeneration through the induction of a transcript level of *MDHAR*, and of DHAR in rice cultivars under saline or non-saline stress conditions. Of course, there are other genes encoding MDHAR and DHAR in plants^42^ that require further study under similar conditions.

In general, roots are able to regulate/restrict ion movement through the presence of physical barriers such as endodermal and in some cases peridermal and exodermal layers^79–81^. By altering the chemical properties of these barriers, plants are able to enhance molecule selectivity and block the uncontrolled movement of ions forcing them through a symplastic pathway. Strengthening the endodermal and peridermal cell walls has been related to increased salinity tolerance^82^. Consistent with the physiological adaptations, we also found structural modifications in the root anatomy of rice cultivars under different treatment regimes. Callose plays an important role in plant development and defense related responses under different abiotic/biotic stress conditions^83^. The deposition of callose in roots is considered to be the most sensitive indicator of stress from heavy metals/salinity^84, 85^ (Fig. 4). Increased production of callose under saline stress might be due to the improved availability of Na^+^ in the root system, which may lead to the disruption of the plasma membrane through lipid peroxidation, which consequently raises the Ca^2+^ penetration into cells and trigger a temporary increase of callose production^82^. In contrast, the accumulation of Na^+^ in the cell walls may induce the indirect synthesis of callose through the disruption of homeostasis between the cell wall and cytoplasmic membrane, as observed in the case of Al^3+^ toxicity^86, 87^. In this way, callose deposition play an important role in the defense response in rice roots as an effective barrier that can markedly limit the amount of Na^+^ that enters the protoplasts^88^. Furthermore, the enhanced accumulation of callose under combined treatments may be due to the accumulation of ABA, because it also regulates callose deposition under stress conditions. In this study, a higher deposition of callose in the XS 134 cultivar (Fig. 4) under saline stress and combined stress treatments may act as an element of defense against the penetration of toxic Na^+^ in root cells^89^. In addition to callose modifications, rice roots undergo extensive lignin deposition changes, such as the intense lignification of endo and exodermises to create tight barriers as an adaptation mechanism under saline stress and combined stress conditions^90, 91^.

An increase in lignin synthesis and its deposition in root cells contribute significantly to the improvement of plant resistance to the toxic effect of salinity^92^. The control and expression of lignin genes are under the influence of H_2_O_2_ production, ABA concentration, POD and PAL activities^93, 94^. In the present investigation, the sensitive cultivar accelerated a relatively higher deposition of lignin in the roots under saline stress conditions compared to the resistant cultivar; this might be due to the increased activity of POD and H_2_O_2_ production (Table 2) in the sensitive compared with the resistant cultivar (Fig. 5). However, under combined stress treatments, lignin deposition was relatively higher in the resistant cultivar as compared to the sensitive cultivar (Fig. 5). This is likely due to 2,4 D accelerated ABA production which triggers key enzymes of lignin biosynthesis (PAL and POD) in the resistant cultivar compared to the sensitive one^94^. This phenomenon can also be observed in 2,4-D treated roots, where lignin deposition was accelerated under the influence of herbicide additions in the growth media compared to the control (Fig. 5b and f). These findings suggest that superior stress tolerance in the XS 134 cultivar is associated with its root anatomical traits (Figs 5 and 6). The ability of roots to deposit lignin and callose is a vital anatomical adaptation in plants that allows plants to grow under stressful conditions^64, 65, 68^. However, these root anatomical traits alone were not sufficient to confer saline/herbicide tolerance, but could explain the different adaptation strategies of plants under individual and combined stress conditions.

The present results indicate that the root biomass exhibited a decreasing trend, when the NaCl concentration in solutions increased^9^. Meanwhile, significant genotypic differences among rice cultivars were observed at each saline level. The XS 134 cultivar, a saline tolerant cultivar, had the highest root biomass compared to the sensitive cultivar ZJ 88, which showed lower root biomass under mild and severe saline treatments, respectively. Furthermore, the 2,4-D treatment alone significantly decreased the root biomass in ZJ 88 compared to XS 134. It has been established that exogenous auxin application triggers the conjugation of endogenous IAA with aspartate, thereby causing a decrease in the endogenous IAA level^95^, which may be the main reason for the reduced root biomass in rice cultivars under 2,4-D treatment alone. Filin and Ivanov^96^ also observed that a higher concentration of 2.4-D inhibits the growth of *Arabidopsis* roots due to the prolongation of the cell cycle and life span of the cells in the meristem and decreases cell elongation and proliferation^97^. Interestingly, the recommended dose of 2,4-D additions, significantly alleviated the inhibition of root growth caused by saline stress in both cultivars differentially (Table 1). Previously, Gulnaz *et al*.^98^ also demonstrated an increase in root biomass in 2,4-D primed wheat plants under saline stress conditions.

To elucidate the molecular mechanism of salt tolerance under saline stress and 2,4-D application, the expression of different salinity responsive ion transporters were monitored in the roots of both cultivars. The *OsHKT1;5*, which is localized in the xylem parenchyma cell root, restricted further Na^+^ translocation from the root to shoot by mediating the xylem Na^+^ from unloading in the roots^98^. The activity of *HKT1;5* in the roots prevents the accumulation of sodium in the sensitive tissue of plants, such as leaves. The induced expression of *OsHKT1;5* was shown to be associated with reduced Na^+^ accumulation in the roots^99^, as observed in the present investigation, where an induced expression of *OsHKT1;5* was observed in cultivar XS 134 compared to the cultivar ZJ 88 under saline alone and combined stress conditions (Fig. 3). Previously, an increase in the activity of *OsHKT1;5* has been observed in resistant cultivars, whereas reduced expression was noted in the sensitive cultivar (Pokkali and FL478)^100^. Thus, it is possible in the present study that the improved root growth of the XS 134 cultivar may have been due to the expression of *OsHKT1;5*, whereas less biomass production in the sensitive cultivar may be associated the downregulation of *OsHKT1;5* expression, resulting in unregulated Na^+^ uptake and impairment transport to the leaves and reduced growth^101^.

Plasma membrane proteins 3 (*PMP3*) are a class of small, conserved, hydrophobic proteins that are induced in response to a wide variety of abiotic stress conditions, thus suggesting their role in membrane stability^102^. The deletion of *PMP3* in yeast increased Na^+^ and K^+^ sensitivity and resulted in the excessive accumulation of Na^+^ and K^+^^102^. The expression of *PMP3* homologues, such as *Oslti6a* and *Oslti6b* in rice, *wpi6* in wheat, *AcPMP3-1* and *AcPMP3-2* in grass and *ZmPMP3* genes in maize, contributed to the regulation of intercellular ion homeostasis and prevent excessive accumulation of Na^+^ influx^103, 104^. In the present study, the expression of *PMP3* orthologs (Oslti6a and Oslti6b) showed differential expression patterns in the root of cultivars under saline and combined stress conditions, which suggested the involvement of these genes in the regulation of Na^+^ entry in the rice cultivars, whereas downregulation under saline conditions may be the reason for the higher accumulation of Na^+^ (Fig. 3 and Table 1). Fu *et al*.^105^ suggested that PMP3 proteins effect the expression of some other ion transporter gene through the regulation of membrane potential, which helped in the maintenance of intracellular ion homeostasis in plant roots^106^.

Another factor that may contribute in the adaptation to the saline stress and less Na^+^ uptake in the XS 134 cultivar might have been related to the expression of *OsCNGC1*. The non-selective cation channels such as cyclic nucleotide-gated channel 1 (*OsCNGC1*) gene transporter mainly function in uptake of Na^+^ into the root cells^107^ (Fig. 3). Recently, enhanced expression of *OsCNGC1* in roots was found in the sensitive cultivars (IR29 and Sakha), while downregulation was observed in the resistant cultivars (Pokkali and Egyptian Yasmine) under salinity^107^. The induction of the *OsCNGC1* gene in the ZJ 88 cultivar showed the existence of facilitated Na^+^ uptake route in plants; this may explain the higher accumulation of Na^+^ in the roots of ZJ 88 cultivar under both saline and combined treatments. Conversely, it’s lower expression level under saline and combined stress treatments in the XS 134 cultivar suggest dynamic control on Na^+^ uptake at root level. Another gene/transporter, *OsHKT2;1* have been reported to be involved in mediating Na^+^ uptake from the soil under K^+^ deficient conditions. A mutant of the *OsHKT2;1* showed reduced Na^+^ accumulation, but no change in the K^+^ accumulation, which suggest that *OsHKT2;1* contributes to Na^+^ uptake in a K^+^ availability-dependent manner^108^. Under saline conditions, an expression of *OsHKT2;1* was enhanced in the ZJ 88 cultivar, while under combined treatment its expression was downregulated in both cultivars (Fig. 3). Previously, Horie *et al*.^109^ found that *OsHKT2;1* was reduced in the rice plants under saline stress conditions which may restrict the accumulation of Na^+^ in rice plants. Conversely, its induction in the ZJ 88 cultivar may be the reason of enhanced accumulation of Na^+^ in roots or may be associated with increased Na^+^ translocation into the shoot, instead of reducing Na^+^ uptake^110^. However, under combined stress condition, its expression was downregulated and less Na^+^ accumulation was recorded (Fig. 3).

Salt overly sensitive 1 (*SOS1*) is a Na^+^/H^+^ exchanger and well-reported salt stress tolerance related protein. *SOS1* is primarily involved in optimizing cytosolic ion homeostasis by transporting Na^+^ out of the cell under saline stress condition^111, 112^. A *sos1* mutant of *Arabidopiss* demonstrated that *SOS1* is involved in ion efflux from the cytosol to the surrounding medium in epidermal cells and to the vascular tissues from surrounding parenchyma, thus maintaining low concentrations of Na^+^ in root cells^112^. The upregulation of *OsSOS1* can immediately decrease Na^+^ content in roots because their upregulation increased the frequency of Na^+^ exclusion from root epidermal cells into the rhizosphere. In this study, we also found a considerable induction of *SOS1* transcript abundance under the saline stress conditions (Fig. 3). However, under combined treatment more strong induction was observed in the ZJ 88 cultivar roots, which is correlated with high Na^+^ accumulation in the roots (Table 1). This might be explained by the need of stressed plants to pump more Na^+^ out of the cell, either via loading of Na^+^ into the xylem or via extrusion of Na^+^ back to the growth medium^113^.

Na^+^ vacuolar compartmentalization through Na^+^/H^+^ antiporter (*NHX1*) reduces Na^+^ concentration in cytoplasm and osmotic potential in the vacuoles^87, 114^. The activity of Na^+^/H^+^ antiporter (*NHX1*) in stressed cell relieves the toxic effect of excess Na^+^ accumulation on cytosolic enzymes and enhances salt tolerance by affecting the physiological processes through the regulation of pH and K^+^ homeostasis^115, 116^. Previously, a strong correlation among the expression of *NHX1* antiporter gene and salt tolerance was observed, which suggest that induced transcript abundance in the roots of the resistant cultivar facilitate Na^+^ exclusion from cytosol into the vacuoles^117^. In the present investigation, higher transcript expression was also found in the roots of the sensitive cultivar (Fig. 3) that may be the adaptive strategy to combat with excess Na^+^ through its sequestration into the vacuoles^117^. However, the transcript expression was decreased in sensitive cultivars under combined stress conditions, while no change in the expression was noticed in resistant cultivar compared to their respective saline stress treatment. These results are in lined with the previous result of Mekawy *et al*.^101^, who found enhanced expression of *OSNHX1* in the sensitive cultivar under saline stress and suggested that induced expression of *OsNHX1* would be mainly in response to the elevated Na^+^ levels in the sensitive cultivar (Table 1).

*OsAKT1* is mapped on the chromosome 1 and is located in epidermis, cortex, and endodermis of the root^118, 119^. The *AKT1* is a major channel for uptake of K^+^ from the soil. Under saline stress conditions, AKT-type K^+^ channels formed by *AKT1* protein are downregulated, which hyperpolarizes the plasma membrane due to the lack of positively charged K^+^^120, 121^. This enhanced hyperpolarization of plasma membrane increases the influx of Na^+^ thorough other routes like non-selective cation channels (CNGC), which result in the increased Na^+^ accumulation and induced salt sensitivity^118^. The expression of *OsAKT1* was decreased under saline stress in the ZJ 88 cultivar, while in combined treatments transcript abundance of *OsAKT1* was enhanced in both cultivars (Fig. 3). The superior maintenance of transcript expression in cultivar XS 134 would explain the increased K^+^ accumulation in roots as compared to the ZJ 88 cultivar (Table 1). The expression of *OsHAK7*, low-affinity K^+^ transport complementing potassium channels^122, 123^, was significantly upregulated in the sensitive cultivar as compared to the resistant cultivar, which may have resulted in the excessive accumulation of Na^+^ in the ZJ 88 cultivar roots and low Na^+^ levels in the XS 134 cultivar roots (Table 1). These results are in lined with the studies of Mekawy *et al*.^101^, Senadheera *et al*.^107^, who found that the upregulation of *OsHAK7* in the sensitive cultivar, while relative less induction may help to reduce overall load of Na^+^ accumulation in the resistant cultivar compared with sensitive cultivar (Fig. 3). However, *OsHAK7* downregulation under hormone treatment might be useful for the K^+^ supply of the plants^124, 125^. The results of cations transporter proteins showed that application of 2,4-D under saline stress conditions had a significant effect on salt tolerance in rice by dynamic regulation of Na^+^ and K^+^, although the responses varied between two cultivars owning to differential salt sensitivity. However, the mechanisms of differential regulation of ion transporters under 2,4-D application is not clear, which requires further study.

### Possible mechanisms of salt tolerance

**Figure 7:**
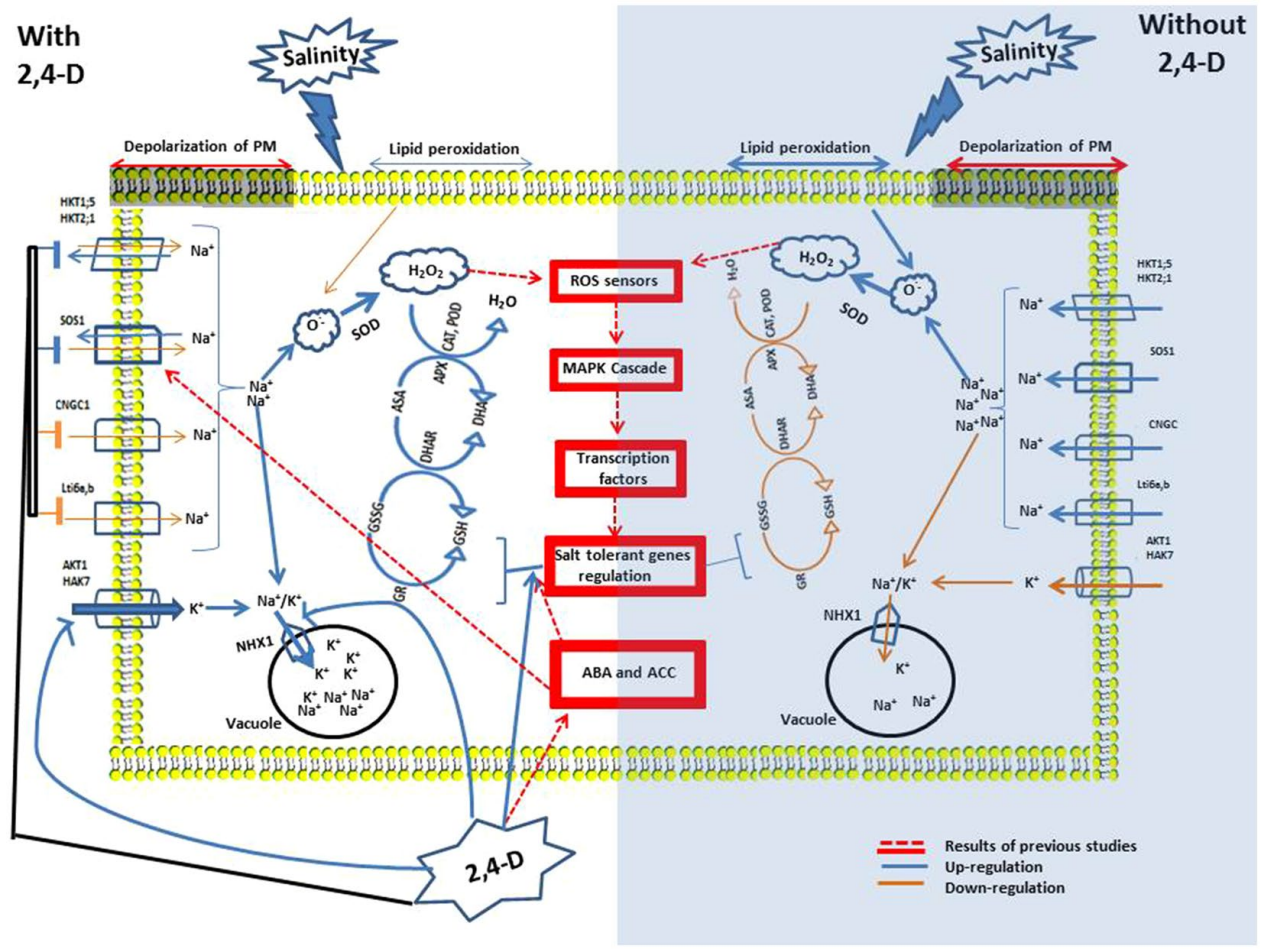
A schematic model based on the present study for plant defense responses under salinity with or without 2,4-D application. Under saline stress conditions, Na^+^ passes through the cell membrane via nonselective cation channels (*NSCC*), induces membrane depolarization, which deactivate potassium (K^+^) inward rectifier channels (*AKT*) that limits plant growth by interfering with major physiological functions, disrupting ion homeostasis, diminishing nutrient uptake and produce excessive ROS and lipid peroxidation due to enhanced cellular Na^+^ level. 2,4-D application modulates the expression of cation transports proteins and antioxidant defense system to reduce ROS production and Na^+^  uptake in cell through the activation of the salt overly sensitive (*SOS*) signaling pathway and compartmentalization of sodium in vacuole by Na^+^/H^+^ exchangers (*NHX*).

Till now, limited information is available about how 2,4-D is involved in regulating stress responses in plants. In the present investigation, we demonstrated that 2,4-D can mediate antioxidant defense and Na^+^/K^+^ transporters to improve salt tolerance in rice roots. However, the precise mechanism by which 2,4-D regulates K^+^ uptake and the K^+^/Na^+^ balance in rice roots remains unclear. In this manuscript, we have presented the differences in salt sensitivity between two contrasting rice cultivars under 2,4-D application. We have analyzed several parameters associated with salt stress responses in both cultivars and their differences may underlie their differential salt tolerance under individual and combined stress conditions (Fig. 7). It is well established that salinity causes depolarization of the plasma membrane, which allows the entry of Na^+^ through CNGC into the cytoplasm. Therefore, the excessive Na^+^ restricts the assimilation of K^+^ because of its competition for the sites of K^+^ uptake^126^. Moreover, salinity induced elevation in cytosolic Ca^+^^127^ and its subsequent activation of NADPH oxidase results in a dramatic promotion in ROS^128^, leading to the protein degradation and lipid peroxidation. On the other hand, 2,4-D activates the antioxidant defense to scavenge the ROS production and prevent the oxidation of vital organelles and membranes under salt stress. Additionally, due to persistence nature of 2,4-D compared to the natural auxins, it stimulates the synthesis of stress hormones ABA and ethylene^129^. The higher concentration of ABA regulates Na^+^/H^+^ antiporter (*SOS1*)^130, 131^, which may efflux Na^+^ out of cytoplasm and decrease salt induced depolarization of plasma membrane which limits the K^+^ efflux. Furthermore, enhanced activity of the PM H^+^-ATPase due to the ethylene, regulates the Na^+^/H^+^ antiporter to sequester Na^+^ into the vacuoles^132^. As a result, lower Na^+^ concentration in the cell allows the *HAK7* and *AKT*1 to uptake K^+^ (which was suppressed due to higher concentration of Na^+^ inside), leading to higher K^+^ concentrations and K^+^/Na^+^ ratio in the cytoplasm of salt-treated rice roots. Taken together, one of the salt tolerance mechanisms may be related to the tight regulation of Na^+^ induced ROS production through the upregulation of antioxidant system. A second tolerance mechanism is associated with the efficient control of Na^+^ transport through the HKT transports. A third tolerance strategy is the compartmentalized of Na^+^ into vacuole through the NHX antiporters. Furthermore, strengthening of cell wall with callose and lignin may restrict the apoplamic flow of Na^+^ that also helps in the salt tolerance.

## Conclusion

It is concluded that application of 2,4-D acts as a salt protectant and promotes rice growth by modulating the cation regulation and anatomical changes. Here, the possible mechanism in the improvement of all these major metabolic activities could be associated with the known role of auxin in the cell division, boasting of enzymatic and non-enzymatic activities and regulation of mineral nutrients under stress conditions. Conversely, the 2,4-D application alone also produces oxidative stress and lipid peroxidation in rice cultivars. However, under saline stress conditions, 2,4-D significantly improved root biomass, reduced the ROS production and enhanced the antioxidant enzymes, which might be due to the lower accumulation of Na^+^ and higher ration of K^+^/Na^+^ ratio under combined treatments. On the basis of our results, the 2,4-D application in the field can attenuate saline stress in rice plant depending upon severity of saline stress and crop sensitivity to the 2,4-D application. However, further studies are needed to elucidate the complicated interplay of salinity and 2,4-D at the molecular level.

## Materials and Methods

### Plant material and experimental design

The healthy and uniform seeds of two rice (*Oryza sativa* L.) cultivars i.e. Zhejing 88 (ZJ 88) and Xiushui 134 (XS 134) were surfaced sterilized in 0.1% NaClO for 15 min, then rinsed and soaked with distilled water for further 20 min. The seeds were germinated on moistened filter paper kept in darkness for 48 hr, and then in a growth chamber with day/night temperatures of 24/16 °C, a 16-hr photoperiod, irradiance of 300 μmoL m^−2^ s^−1^, and relative humidity of 60–70%. Ten days old uniform seedlings were transferred to greenhouse in plastic pots containing 5 L of half strength nutrient solution. After one week of acclimatization, plants were treated with 4 dS m^−1^ and 8 dS m^−1^ concentrations of NaCl based upon our previous study^10^. Usually salinity is measured in units of electrical conductivity (EC), and according to the International Rice Research Institute (IRRI) salinity beyond ECe~4 deciSiemens per meter (dS m^−1^) is considered as moderate salinity while more than 8 dS m^−1^ becomes high for rice plants. The herbicide 2,4-D can be directly applied onto the soil in the form of granules or mixed with sand for easy operation according to IRRI guidelines^133–135^. To mimic field conditions, we added recommended dose of 2,4-dichlorophenoxyacetic acid (0.8 kg a.i. ha^−1^) in the nutrient solution^133, 134^. Since roots are in direct contact with the soil, it may be expected that they display the first adaptations to cope with increments of salinity/herbicide. To study these internal root tissue adaptations in responses to salinity/herbicide stress hydroponic conditions were selected in order to avoid further external interferences. The experiment was comprised of following treatments: control (CK) with EC 1.2 dS m^−1^, T_2_ (recommended dose of 2,4-D), T_3_ (4 dS m^−1^), T_4_ (8 dS m^−1^ T_5_ (4 dS m^−1^ + recommended dose of 2,4-D), T_6_ (8 dS m^−1^ + recommended dose of 2,4-D). Each treatment was replicated four times. The nutrient solution was renewed every four days. Fifteen days after treatment, samples for morphological, biochemical and expression analyses^44, 136^ were collected as described below.

### Morphological parameters

After 15 days of treatment, plants were harvested and separated into leaves and roots. Root length, and the fresh weight of plants were measured immediately after harvesting; for dry biomass, plants were placed in an oven at 80 ± 1.5 °C for 5 days^137^.

### Determination of malondialdehyde and reactive oxygen species contents

Lipid peroxidation was determined in terms of malondialdehyde (MDA) contents by following the method of Zhou and Leul^138^. Hydrogen peroxide (H_2_O_2_) contents were measured according to the method of Velikova *et al*.^139^. Briefly, fresh leaves (0.5 g) were extracted with 0.1% (w/v) TCA (5.0 mL) in an ice bath and the extraction was centrifuged for 15 min at 12,000 rpm. The supernatant (1.5 mL) was collected after the centrifugation and mixed with 0.5 mL of 10 mM potassium phosphate buffer (pH 7.0) and 1 M KI (1 mL). The H_2_O_2_ contents were calculated by using a standard curve after getting the absorbance of the samples at 390 nm. The superoxide anion (O_2_^−^) levels were determined spectro-photometrically by measuring the reduction of nitro blue tetrazolium (NBT) according to the method of Doke^140^.

### Histochemical staining and electrolyte leakage estimation

The superoxide anion (O_2_^−^) and hydrogen peroxide (H_2_O_2_) production was assayed respectively through nitro blue tetrazolium (NBT) and 3,3′-diaminobenzidine (DAB) staining method as describe by Jambunathan^141^. Histochemical detection of lipid peroxides was done in roots as described by Pompella *et al*.^142^. The roots were stained with Schiff’s reagent for 20 min, which detects aldehydes that originate from lipid peroxidation. After the reaction with Schiff’s reagent, roots were rinsed with sulfite solution (0.5% (*w*/*v*) K_2_S_2_O_5_ in 0.05 M HCl). The stained roots were kept in sulfite solution to retain the staining color. Stained roots were then examined under a light microscope. Electrolyte leakage was measured using electrical conductivity meter as described by Islam *et al*.^143^. The electrolyte leakage (EL) was expressed following the formula; EL = EC1/EC2*100.

### Biochemical analysis

Fresh leaves (0.5 g) were ground in liquid nitrogen with mortar and pestle and then homogenized in 10 mL of ice cold potassium phosphate buffer (pH 7.0). The mixture was centrifuged at 4 °C for 20 min at 12,000 rpm. The supernatants were then stored at −20 °C and used for the determination of various antioxidant enzymes. CAT activity was immediately determined in the supernatant according to Aebi^144^. POD activity was determined as described by Rao *et al*.^145^, ascorbate peroxidase activity was determined according to the method of Nakano and Asada^146^, and SOD activity was assayed as described by Dhindsa and Matowe^147^. One unit of enzyme activity was defined as an absorbance change of 0.01 units per minute and each enzyme’s activity was expressed as unit per milligram protein. Protein content of samples was determined by the Bradford method^148^.

### Determination of non-enzymatic antioxidants

Reduced glutathione (GSH) was analyzed according to Law *et al*.^149^. For this, leaves samples (0.5 g) were homogenized with 10% (w/v) TCA (5 mL) and centrifuged at 15,000 rpm for 15 min. For oxidize glutathione content (GSSG) analysis, 150 µL supernatant was added to 100 µL of 6 mM DTNB, 50 µL of glutathione reductase (10 units mL), and 700 µL of 0.3 mM NADPH. The values of total GSH and GSSG for each sample were calculated from the standard curve generated with known amounts of GSSG. The level of GSH for each sample was obtained by subtracting the GSSG level from the total GSH. All the reagents were prepared in 125 mM NaH_2_PO_4_ buffer, containing 6.3 mM EDTA, at pH 7.5.

GR (EC 1.6.4.2) activity was assayed spectrophotometrically as described^150^. The reaction mixture contained 50 mM Tris-HCl buffer (pH 7.5), 5 mM MgCl_2_, 0.2 mM NADPH, 1.0 mM oxidized glutathione (GSSG), and 0.1 mL of enzyme extract for a final volume of 3 mL. The reaction was initiated with the addition of GSSG, and the decrease in absorbance at 340 nm (due to NADPH oxidation) was recorded for 60 s. Ascorbic acid (AsA) contents of rice roots were estimated following the method described by Mukherjee and Choudhuri^151^. Fresh leaf material (0.25 g) was homogenised in 10 mL solution of 6% TCA. Four ml of the extract were reacted with 2 mL of 2% dinitrophenyl hydrazine solution in the acidic medium. One drop of 10% thiourea solution (prepared in 70% ethanol) was added to the mixture. The mixture was boiled for 20 min in a water bath. After cooling the mixture at 25 °C, 5 mL of 80% H_2_SO_4_ (v/v) were added to the mixture. The absorbance was measured at 530 nm. The amount of AsA in the extracted leaf samples was worked out from a standard curve prepared using varying AsA standards. Monodehydroascorbate reductase activity was determined by measuring oxidation of NADH at 340 nm according to the Hossain^152^. The dehydroascorbate reductase activity was measured at 265 nm by the method of Dalton *et al*.^153^. The reaction mixture contained PBS (1.5 mL), EDTA (300 μL), GSH (500 μl), DHA (300 μL) and enzyme extract (400 μL).

### Visualization of callose and lignin

For callose determination, root tips (0.5 cm) were dipped into 85% (v/v) ethanol and incubated for over 12 h at room temperature (25 °C) with gentle shaking. Then roots were placed into 1% (w/v) aniline blue solution in 1 M glycine (pH 9.5) for 5 h at RT and thoroughly washed with double distilled water^154^. The cross-sections of root segments, which were stained with aniline blue, were observed under a fluorescence microscope (NIKON ELIPSE N*i* equipped with LEICA DFC 425 camera) with UV illumination.

Five mm root pieces were taken from the middle of the root zone. Root pieces were immersed in saline phosphate buffer (pH 7.4) containing formaldehyde (3.7%; w/w)^155^. To detect lignin in the root, root cross sections were stained for several minutes with the aid of the Maule reaction^156^, which gives different colors depending on the composition of lignin monomers^157^. Lignified tissues were stained orange/brown staining under white light. The experiment was replicated four times for each treatment.

### Determination of Na^+^, K^+^ and lignin

For Na^+^/K^+^ determination, 50–100 mg of dry matter of each sample was subjected to an overnight digestion with HNO_3_/H_2_O_2_ according to method described by Munns *et al*.^158^. The content of Na and K was determined using atomic absorption spectrometry.

Lignin was quantified according to the method of Ma *et al*.^159^. Roots (0.5 g) were homogenized in 95% ethanol and centrifuged at 1,000 g for 10 min. The supernatants were then discarded, and the pellets were washed with 95% ethanol: chloroform (1:2). The segments were then dried at 50 °C and digested in 0.5 ml of 25% (v/v) acetyl bromide in acetic acid at 70 °C for 30 min, cooled quickly, then added to 0.9 ml of 2 M NaOH to terminate the digestion. The samples were then mixed with 0.1 ml of 7.5 M hydroxylamine hydrochloride and 5 ml of acetic acid and centrifuged at 1,000 g for 5 min. The amount of lignin (mg of protein) was determined based on the absorbance at 280 nm, as mean values of four replicates ± standard error.

### RNA isolation, cDNA synthesis and real-time RT-PCR assay

Total RNA from roots of rice cultivars under different treatment regimens after 15 days of stress treatments (as described earlier), were isolated with TaKaRa MiniBEST plant RNA extraction kit (Takara Bio Inc., Japan) according to the manufacture protocol. Integrity of isolated RNA samples was analyzed through spectrophotometrically and by gel electrophoresis. For real time RT-PCR analysis, 1 μg of total RNA was reverse transcribed by using the primer Script RT reagent Kit with gDNA Eraser (Takara Bio Inc., Japan). cDNA samples from different treatments were assayed by qRT-PCR in the iCycleri QTM Real-time detection system (Bio-Rad, Hercules, CA, USA) using SYBR® Premix Ex Taq II (Takara Co. Ltd). The iCycler IQ Real-Time Detection System Software was used to calculate the threshold cycle values and quantification of mRNA levels was calculated according to the method of Livak and Schmittgen^160^. Primers used for the amplification of target cDNAs were designated according to rice genome available in the databank (http://www.ncbi.nlm.nih.gov/). The specific primers used for each gene are presented in Supplementary Table 1.

### Ultrastructural observations

For electron-microscopic study, root tips (about 2–3 mm) were fixed in 2.5% (v/v) glutaraldehyde in 0.1 M sodium phosphate buffer (pH 7.4) overnight and then washed three times with buffer. The samples were post fixed in 1% (m/v) OsO_4_ for 1 h and washed again three times with buffer. Thereafter, the samples were dehydrated in a graded series of ethanol (50, 60, 70, 80, 90, 95, and 100%, v/v) for 15–20 min each and then in absolute acetone for 20 min. After dehydration, the samples were embedded in Spurr’s resin overnight and heated for 9 h at 70 °C. After that ultra-thin sections (80 nm) were cut and mounted on copper grids for transmission electron microscopy (TEM 1230EX, JEOL, Japan) at 60 kV. Three samples per treatment were used for ultrastructural observations studies.

### Statistical analysis

All the treatments were arranged in a completely randomized block design. Morphological and physiological/biochemical data were presented as mean values of four replicates ± standard error. The data were analyzed using a statistical package, SPSS (Version 19.0). One-way analysis of variance was employed followed by Duncan’s multiple range test to determine the significant differences among means of the treatments at 5% level of significance. For gene expression data (three replicates ± standard error), two-way analysis of variance was employed followed by Duncan’s multiple range test to determine the significant differences among means of the treatments at 5% level of significance.

## Electronic supplementary material

Supplementary information
