# Supplementary material for: 2,4-D attenuates salinity-induced toxicity by mediating anatomical changes, antioxidant capacity and cation transporters in the roots of rice cultivars
Source: Sci Rep. 2017 Sep 5;7:10443. doi: 10.1038/s41598-017-09708-x (PMC5585390; doi:10.1038/s41598-017-09708-x)
Supplement: Supplementary file 1 — Supplementary information [file 41598_2017_9708_MOESM1_ESM.doc]

**2,4-D attenuates salinity-induced toxicity by mediating anatomical changes, antioxidant capacity and cation transporters in the roots of rice cultivars**

Faisal Islam ^a^, Muhammad A. Farooq ^a,b^, Rafaqat A. Gill ^a^, Jian Wang ^a^, Chong Yang ^a^, Basharat Ali ^a,c^, Guang-Xi Wang ^d^, Weijun Zhou ^a, *^

^a^ Institute of Crop Science and Zhejiang Key Laboratory of Crop Germplasm, Zhejiang University, Hangzhou 310058, China

^b^ Institute of Pure and Applied Biology, Bahauddin Zakariya University, Multan, Pakistan

^c^ Institute of Crop Science and Resource Conservation, University of Bonn, 53115 Bonn, Germany

^d^ Department of Environmental Bioscience, Meijo University, Nagoya City, Aichi 468-8502, Japan

* Corresponding author. Tel.: +86 571 88982770. E-mail address: [wjzhou@zju.edu.cn](mailto:wjzhou@zju.edu.cn)

**Supplementary table 1**

**Table S1** Sequences of primer pairs used in real-time PCR.

| **Gene name** | **Gene description** | **Primer sequence** |
| --- | --- | --- |
|  |  |  |
| **CAT1** | Catalase isozyme A | 5- TAAGGCCAGACAATGTCAGATG  3- CAGTGGCATTAATACGCCAGTA |
| **CAT2** | Catalase isozyme B | 5- GCACAGTTTGACAGGGAG  3- GGTCTGAACACCAGGAGC |
| **APX1** | Cytosolic ascorbate peroxidase 1 | 5- AGTACATTGCCCGTGGTACTCT  3- CGCATTTCATACCAACACATCT |
| **APX2** | Cytosolic ascorbate peroxidase 2 | 5- GGACCAACTTCCCATCCTC  3- TGTCACTCAAACCCATCTGC |
| **CuZnSOD1** | Cytosolic copper/zinc-superoxide dismutase 1 | 5- TGGCAGAGCCGTCGTTGT  3- CGATGATCCCGCAAGCAA |
| **MnSOD** | Mitochondrial manganese-superoxide dismutase | 5- TTTGGTTCATTTGAGGCACT  3- GTCCTGGTTAGCAGTTGTTTCC |
| **POD** | Peroxidase | 5- TGCTTACCAAGAGCGCTGAA  3- TGATCCAGTCAGAGGCGAGA |
| **GR1** | Cytosolic glutathione reductase | 5-TATCCACGGAAAGGAAACC  3-TCCAGGCTGTGGTACTCAC |
| **GR2** | mitochondrial glutathione reductase | 5-TCCAAACGGCAGGAGAAGACC  3-CAGCAGCAGACGGGTGAATA |
| **DHAR1** | Dehydroascorbate reductase | 5-GACGAAGGCAGCTAAGGA  3-CATGGACCGATCAGATAAAC |
| **MDHAR1** | Cytosolic monodehydroascorbate reductase | CTGGGATGGCAATTCTACGG  TTGGCAATGGCCTTGTTCTC |
| **MDHAR2** | Putative monodehydroascorbate reductase | 5-AGAAGCCTGCTCGTCTACC  3-CATCAAACGCTACAACTGGA |
| **P5CS** | Delta-1-pyrroline-5-carboxylate synthase | CAAATGCTCCTTTTAGCCTGTT  GCGTTGGTACACAAGTTCTCAG |
| **OsHKT1;5** | Sodium transporter Hkt1.5 | 5-CCCATCAACTACAGCGTCCT  3-AGCTGTACCCCGTGCTGA |
| **OsLti6a** | Plasma membrance protein3 | 5-CCTTCCAAGGTGATGGTGAA  3-CCGTCCAAAGAACCAGAAAA |
| **OsLti6b** | Low molecular weight integral membrane protein | 5-GCTCCAAACCGCTTCATCTA  3-CAAGAATTGGAGCACTCAGGA |
| **OsHKT2;1** | High-affinity potassium transporter | 5-TGCATTCATCACTGAGAGGAG  3-GGTGCAGTTTCTGCAACCTC |
| **OsNHX1** | Na+/H+ antiporter | 5-AATGATCACCAGCACCATCA  3-AAGGCTCAGAGGTGACAGGA |
| **OsSOS1** | Na+/H+ antiporter | 5-ATACTGAGTGGGGTTGTTATTGC  3-AAAGGTAAATTTCAAAAGGTACATGG |
| **OsAKT1** | Serine/threonine-protein kinases | 5-GAAACGAGCAATGCGTCAG  3-CTTCTCACACAGCGCTTCC |
| **OsHAK7** | Potassium transporter 7 | 5-TGCTGTGACACTTGGTTTCC  3-AAATAACAAGGCGAGCAGGA |
| **OsCNGC1** | Non-selective cation channels 1 | 5-TGCAATAGCAAAGCGATACTTG  3-TTTTGGCTTTTGCAACCTCT |
| **OsUBQ5** | Ubiquitin 5 | 5-ACCACTTCGACCGCCACTACT  3-ACGCCTAAGCCTGCTGGTT |
